# E3-ligase knock down revealed differential titin degradation by autophagy and the ubiquitin proteasome system

**DOI:** 10.1038/s41598-021-00618-7

**Published:** 2021-10-26

**Authors:** Erik Müller, Senem Salcan, Sabine Bongardt, David Monteiro Barbosa, Martina Krüger, Sebastian Kötter

**Affiliations:** Department of Cardiovascular Physiology, Medical Faculty and University Hospital Düsseldorf, Heinrich-Heine-University Düsseldorf, Universitätsstr. 1, 22.03 02, 40225 Düsseldorf, Germany

**Keywords:** Cardiovascular biology, Autophagy, Post-translational modifications, Proteolysis

## Abstract

The sarcomere protein titin is a major determinant of cardiomyocyte stiffness and ventricular distensibility. The constant mechanical stress on titin requires well-controlled protein quality control, the exact mechanisms of which have not yet been fully elucidated. Here, we analyzed E3-ligases potentially responsible for cardiac titin ubiquitination and specifically studied the involvement of the autophagosomal system in titin degradation. Pharmacological inhibition of autophagy and the proteasome in cultured primary rat cardiomyocytes significantly elevated titin ubiquitination and increased titin degradation. Using *in-vitro* pull down assays we identified binding of E3-ligases MuRF1-3, CHIP and Fbx32 to several titin domains. Immunofluorescence analysis showed sarcomeric localization of the E3-ligases. siRNA-mediated knock-down of the E3-ligases MuRF-1, -3 and a combination of CHIP/Fbx32 significantly reduced autophagy-related titin ubiquitination, whereas knock-down of MuRF-2 and -3 reduced proteasome-related titin ubiquitination. We demonstrated that the proteasomal and the autophagosomal-lysosomal system participate in degradation of the titin filament. We found that ubiquitination and degradation of titin are partially regulated by E3-ligases of the MuRF family. We further identified CHIP and Fbx32 as E3-ligases involved in titin ubiquitination.

## Introduction

The structural integrity and elasticity of cardiomyocytes are of particular importance for the myocardium as they strongly define its diastolic properties. Increased diastolic stiffness of the ventricle has been reported to be a hallmark of heart failure with preserved ejection fraction^1,2^. The overall ventricular stiffness is mainly determined by the combination of extracellular matrix stiffness (e.g. collagen content and cross-linkage), and myofilament passive stiffness and distensibility. The latter is mediated by the filament protein titin. Together with actin and myosin filaments titin forms the structural backbone of the sarcomeres of striated muscle cells. The elastic properties of titin are mediated by serially linked extensible I-band domains that define titin’s function as a molecular spring and determine cardiomyocyte passive stiffness^3^. In cardiac muscle, titin exists in two isoform types, which are co-expressed in a half sarcomere: the longer and more compliant N2BA isoforms (~ 3.2–3.3 MDa) and the shorter and stiffer N2B isoform (~ 3.0 MDa). The ratio of sarcomeric N2BA and N2B isoforms strongly determines titin-based passive stiffness^4^ and can be modified in order to adapt myocyte stiffness to altered mechanical demands of the myocardium. During perinatal heart development, titin isoform expression shifts from fetal N2BA isoforms to adult N2BA and N2B isoforms^5^. In the adult heart titin isoform switching was reported for several types of heart failure^6^.

The half-life of a titin molecule is only about 3–5 days^7^ and its gigantic size makes it highly susceptible to fragmentation and degradation. This indicates that a continuous and well-defined protein quality control (PQC) is necessary. PQC systems generally control the fine-tuned balance of protein turnover and synthesis. The three main components are the ubiquitin proteasome system (UPS), the autolysosomal system and the calpains^8^. Proteases like calpains are particularly important for the pre-digestion of filament proteins, including titin, because they are required to make the substrates accessible for the following ubiquitination process^9^. The UPS is a central part of the PQC and responsible for the regulated non-lysosomal degradation of the majority of misfolded or defective cytoplasmic proteins^10^. The 26S proteasome is built up by a 20S catalytic core and one or two 19S regulatory subunits^11^. The degradation is performed by three protease activities: chymotrypsine-like activity (β1-subunit), trypsin-like (β2-subunit) and peptidyl-glutamyl peptide-hydrolyzing or caspase-like activity (β5-subunit)^11^. Target proteins are recognized by ubiquitin binding proteins within the 19S regulatory subunit, unfolded by heat shock proteins and delivered into the catalytic core for degradation^12^. The autophagosomal-lysosomal system processes single proteins, larger protein complexes, aggregates and complete organelles^13^. There are three different types of autophagy: macroautophagy, chaperone-mediated-autophagy and microautophagy. During chaperone-mediated-autophagy and microautophagy substrates are directly transported into the lysosome and degraded. Macroautophagy describes the degradation process of single proteins, protein aggregates, macromolecules or cellular organelles, and involves encapsulation in a double-membraned vesicle called the autophagosome. In the following macroautophagy will be referred to as autophagy.

Proteins determined for degradation by the proteasome or autophagy are marked by polyubiquitin chains^12,13^. Although ubiquitin molecules in polyubiquitin chains can be interconnected via several other lysine-residues it is commonly accepted that for autophagosomal-lysosomal degradation proteins are mainly marked by polyubiquitin chains interconnected via lysine 63 (K63)^14^, whereas for proteasomal degradation connections are typically formed via lysine 48 (K48)^15^. Ubiquitination is performed in a three-step reaction by E1 (ubiquitin-activation), E2 (ubiquitin-conjugation) and E3-ligases (ubiquitin-ligation). Several muscle specific E3-ligases have been identified, including the muscle specific ring finger (MuRF) family members MuRF-1/2/3, also called tripartite motif containing (TRIM) and the F-box protein Fbx32, also called atrogin-1 or MAFbx^16^. Carboxy terminus of Hsp70-interacting protein CHIP, also called Stub1, is not restricted to but shows the strongest expression in striated muscle. MuRF-2 is important for developmental myofibril assembly in neonatal cardiomyocytes^17^. MuRF-1 and Fbx32 are main regulators of skeletal muscle wasting and Fbx32 is thought to be involved in proteasomal degradation of myosin. In skeletal muscles MuRF-1 and 2 have been suggested to ubiquitinate titin^18^. CHIP seems to be important during ischemia and reperfusion as the absence of CHIP worsens the outcome after myocardial infarction^19^. Direct ubiquitination of cardiac titin by E3-ligases has not been demonstrated yet.

Degradation of the giant protein titin within a working sarcomere is a tremendous challenge for intracellular degradation systems and needs to be precisely controlled. A disturbed turnover of titin could lead to accumulation of non-functional filaments with detrimental effects on sarcomeric functions of the myocardium. It is widely accepted that based on titin’s size a pre-digestion of the titin filament is necessary to release titin from the sarcomeres and to make it accessible for turnover. Candidates for such a pre-digest are calpains^20^ and MMP2 that have been demonstrated to degrade titin in vivo and in vitro^21^. However, although association of autophagy-related proteins to titin has been shown^22^ the role of autophagy in titin degradation is currently unknown. Our group previously reported the involvement of the proteasome in titin degradation^23^. In our present study, we aimed to uncover the participation of the autolysosomal system and to identify E3-ligases that are responsible for titin ubiquitination and subsequent degradation by autophagy or the proteasome.

## Results

### Inhibition of autophagy increases titin ubiquitination in embryonic rat cardiomyocytes

**Figure 1:**
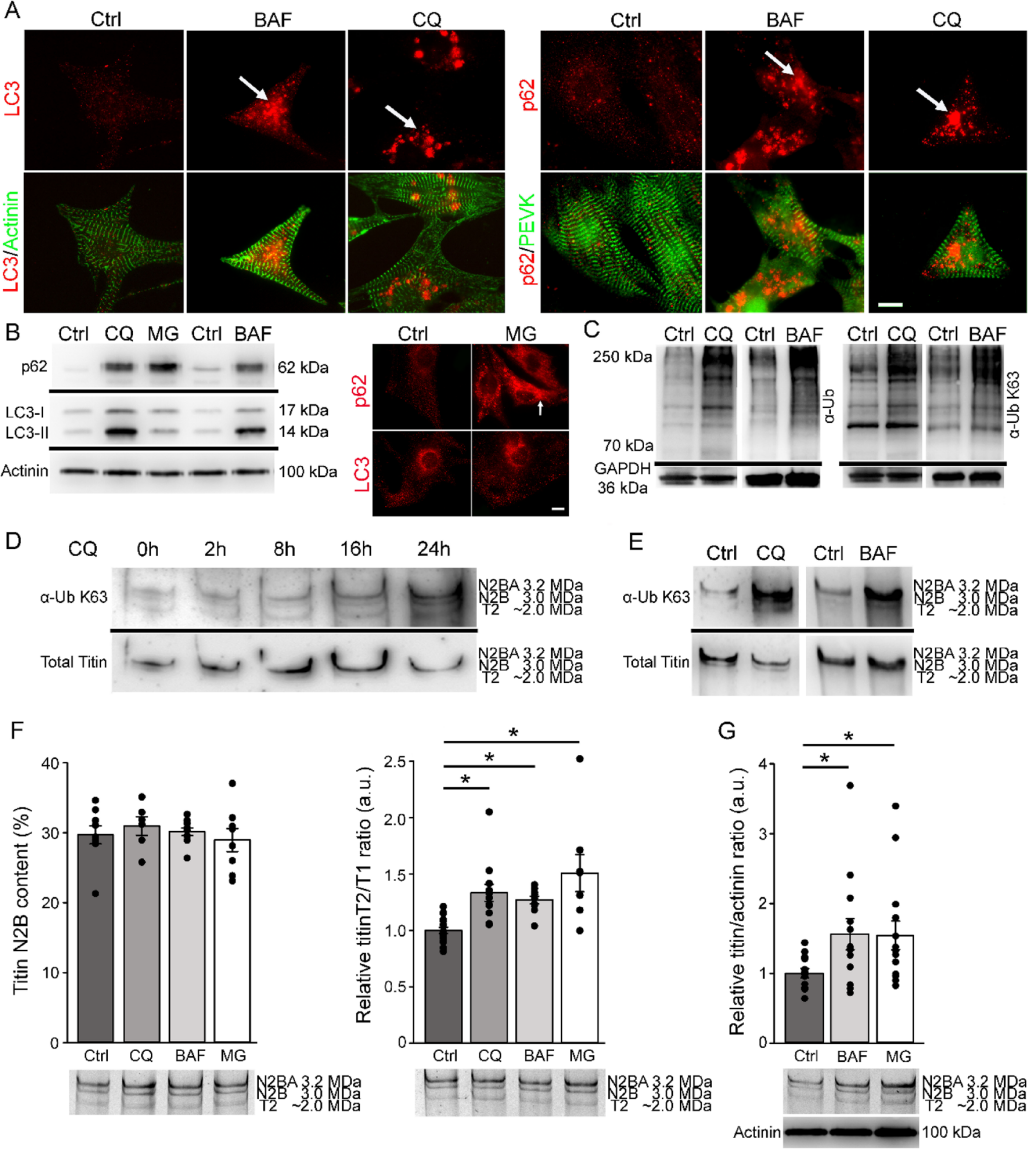
Autophagy and proteasomal inhibition in ERCs induced general protein and full-length titin ubiquitination and increased the level of total titin and titin degradation intermediates. Autophagy related marker proteins LC3 and p62 were investigated under basal conditions and after 24 h of inhibition with CQ, BAF or MG132 by immunofluorescence (**A**/**B**) and Western blot (**B**). (**C**) Protein ubiquitination in the molecular weight range of ~ 70–250 kDa using total ubiquitin and K63-polyubiquitin antibodies. (**D**/**E**) Total ubiquitination and K63-polyubiquitination of titin under control conditions and after autophagy inhibition. (**F**) N2B titin content and relative T2/T1 ratio of ERCs at cultivation day 3 under control conditions and 24 h inhibition of autophagy or the proteasome. (**G**) Relative titin/actinin ratio under control conditions and after autophagy or proteasomal inhibition. An antibody targeting titin PEVK was used in immunofluorescence as cardiomyocyte marker and in titin blot as a marker for titin loading (Total Titin). GAPDH and α-actinin antibodies were used as loading marker for standard Western blots. Data are shown as mean ± SEM (n = 6–17 for T2/T1; n = 13–14 for titin/actinin ratio). Asterisks (*P* < 0.05 in one-way ANOVA with Dunn`s multiple comparison) mark statistical significance. BAF = bafilomycin; Ctrl = control; MG = proteasomal inhibitor MG132; α-Ub = anti-Ubiquitin antibody; α-Ub K63 = anti-K63-polyubiquitin antibody; N2B and N2BA = cardiac titin isoforms; T2 = specific titin degradation band. Bars = 10 µm.

To identify a potential involvement of autophagy in sarcomeric titin degradation we inhibited autolysosomal activity by chloroquine (CQ, 10 µM) or bafilomycin (BAF, 100 nM) application to cultured embryonic rat cardiomyocytes (ERC) and analyzed the effect on the titin filament. To test the efficiency of inhibition by the two substances we performed immunofluorescent stainings and measured the protein levels of two autophagy markers, microtubule-associated protein light chain 3 (LC3 II) and Sequestosome-1 /p62 (SQSTM1, also known as ubiquitin-binding protein p62). LC3-II is widely used to monitor autophagy, as it becomes incorporated into the membranes of maturating autophagosomes^24^ and contains a binding site for SQSTM1/p62, in the following termed p62. P62 is an autophagosome cargo protein that targets other proteins for selective autophagy. In response to autophagy inhibition, both proteins, LC3-II and p62, are expected to accumulate within the cell^25^. In CQ and in BAF- treated ERCs we observed a strong increase in cytosolic LC3 and p62, most likely indicative for non-degraded autophagosomes (Fig. 1A). Under control conditions staining for p62 showed a mainly cytosolic but also some punctate sarcomeric A-band/M-line localization in about 20% of the cells, likely indicating p62 association to titin (Suppl. Fig. 1A)^22^. In addition, both LC3-II and p62 showed strongly increased protein levels in Western blot analysis after 24 h of inhibition by CQ and BAF (Fig. 1B). After proteasomal inhibition with MG132, p62 protein level was elevated and accumulation within cardiomyocytes could be observed, while LC3-II protein level remained unchanged and intracellular LC3 aggregates were not detected (Fig. 1B). We conclude from our observations that the combination of increased LC3 and elevated p62 clearly indicate inhibition of autophagy. To further confirm the effectiveness of CQ and BAF-mediated inhibition of autophagy we analyzed total ubiquitination and K63-dependent polyubiquitination of proteins in the molecular weight range of 70–250 kDa (Fig. 1C) and found markedly increased signal intensities in response to both treatments. Modification of the titin filament was tested using Western blot analyses of agarose-stabilized 2.2% SDS-PAGE, which allowed exclusive detection of titin in the molecular weight range of approx. 3 MDa (Suppl. Fig. 1B). Because no other proteins are known and detectable in this molecular weight range the ubiquitination signals on such blots can be considered to originate from titin. We observed that inhibition of autophagy with CQ for 2–24 h increased the K63-polyubiquitination of full-length titin (Fig. 1D). Incubation for 24 h with BAF resulted in a similar increase of ubiquitinated titin (Fig. 1E). To test if the increased titin ubiquitination is indicative for increased titin degradation, we analyzed the abundance of a specific titin degradation product called T2. This T2 degradation band has been recognized to be a product of the pre-digest and degradation intermediate of titin^6^. As a result of 24 h inhibition of autophagy the levels of the T2/T1 ratio, analyzed by 2.2% SDS PAGE, were significantly elevated (CQ: 33.5% ± 7,6%; BAF: 27% ± 3.3%) (Fig. 1F). A similar increase in the T2/T1 ratio (+ 50.9% ± 16.5%) was observed after blocking proteasomal activity with MG132 (Fig. 1F). After 3 days of cultivation, including 24 h inhibition of autolysosomal or proteasomal activity, composition of cardiac titin isoforms N2B and N2BA remained unchanged compared to control cells (Fig. 1F). To analyze if inhibition of the proteasome or autophagy also alters total titin levels we calculated the titin/actinin ratio. We detected significantly higher levels of titin after proteasomal (+ 56.1% ± 22.4%) or autophagy inhibition (+ 54.3% ± 20.5%; Fig. 1G), compared to controls suggesting accumulation of unprocessed titin.

### E3-ligase interaction with the titin filament

**Figure 2:**
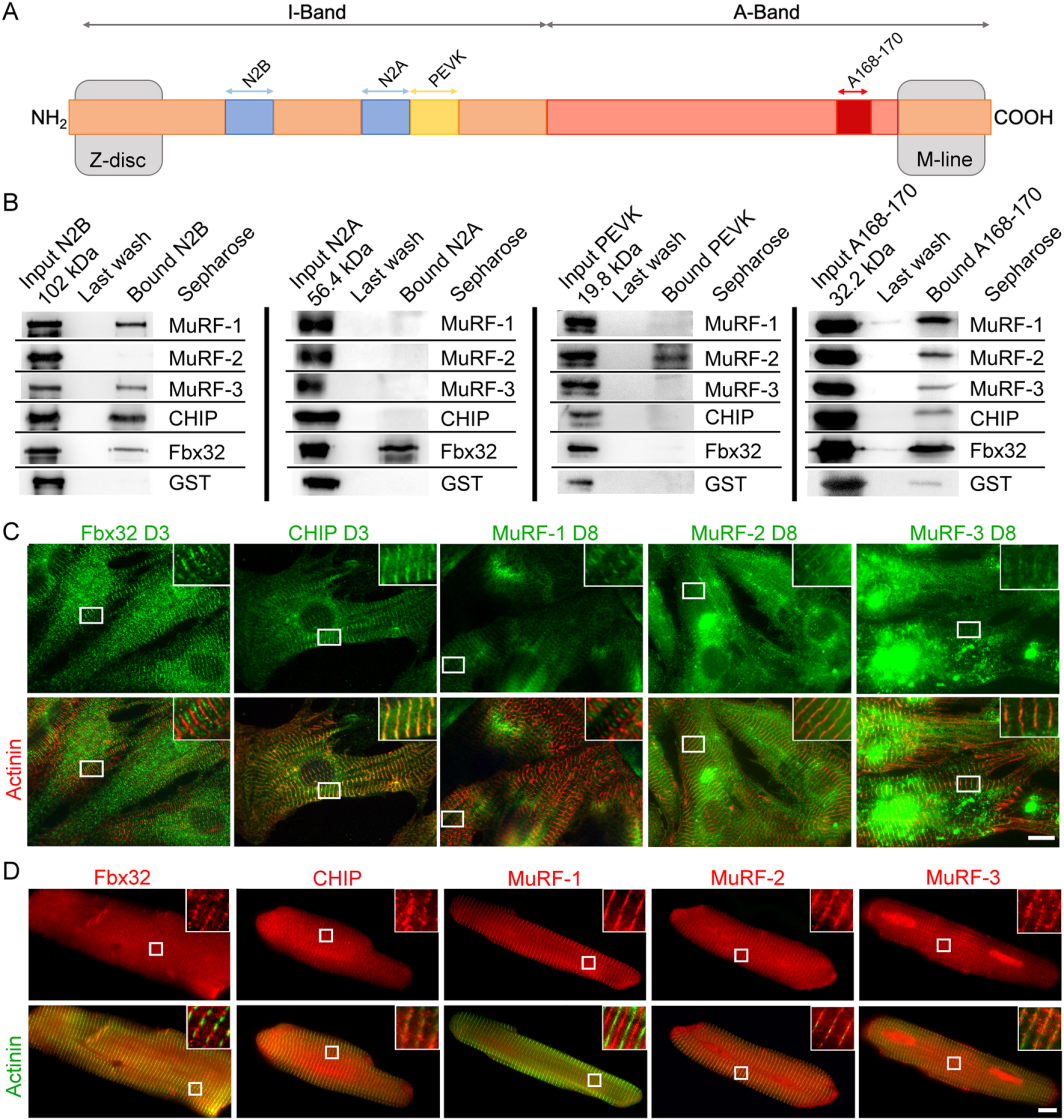
E3-ligase interaction with the titin filament. (**A**) Schematic overview of the titin filament including the analyzed domains, N2B, N2A, PEVK and A168-170. (**B**) In vitro pull-down interaction tests of muscle specific E3-ligases with several titin domains from the I-band and the A-band. Representative images shown for all combinations of interaction tests, including GST controls. Calculated molecular weights of the recombinant titin fragments are shown. Immunofluorescence analysis of E3-ligase localization in (**C**) embryonic and (**D**) adult rat cardiomyocytes under standard cultivation conditions. Insets in **C** and **D** show magnifications of E3-ligase and actinin localization. D3 or D8 = cultivation day 3 or 8. Bar = 10 µm.

Mono- and poly-ubiquitination reactions are performed in 3 steps and are finally catalyzed by E3-ligases. For the ubiquitination reaction, E3-ligases need to be in contact with their target proteins. We therefore tested the interaction of human striated muscle specific E3-ligases, or E3-ligases which are highly expressed in striated muscle, with 4 different human titin fragments by in vitro pull-down assays (Fig. 2A/B). In addition to the previously described titin interaction partners MuRF-1 and 2, we analyzed MuRF-3, CHIP and Fbx32. E3-ligases were immobilized on sepharose beads and incubated with purified titin fragments. All used titin fragments showed a high purity grade (Suppl. Fig. 2A). The affinity of the E3-ligases to the different titin fragments were analyzed by comparison of the input signal to the bound signal (Suppl. Fig. 2B). As the A168-170 fragment showed low binding to the GST-tag control, the GST-bound signal was subtracted for all E3-ligases. MuRF-1 and -3 showed binding to the N2B domain and A168-170. For MuRF-2 we observed similar interaction with the PEVK domain and the A168-170 fragment. CHIP associated to the N2B domain and A168-170 with a trend for higher binding to the N2B domain. Fbx32 interacted with the N2B, A168-170 and the N2A fragment, with the strongest binding to A168-170 (Suppl. Fig. 2B). We further studied intracellular localization of the E3-ligases in cultured ERCs and adult rat cardiomyocytes (ARC). Cardiomyocyte-specific localization was confirmed by co-staining with α-actinin, which is a specific marker for sarcomeric Z-discs in cardiomyocytes. At culture day 3 a subset of ERCs showed sarcomeric localization of CHIP and Fbx32 in the Z-disc/I-band region and A-band, respectively. MuRF-1, -2 and -3 did not show sarcomeric localization after 3 days. However, after 8 days in culture, we detected partial localization of all MuRF isoforms at the A-band (MuRF-1) and the Z-disc/I-band in a small number of cells (< 10%) (MuRF-2 and -3) (Fig. 2C and Suppl. Fig. 3A). In ARCs, all investigated E3-ligases showed sarcomeric localization in almost all cells. In ARC Fbx32 localized to the Z-disc region instead of the M-line, whereas all other E3-ligases had the same localization as seen in ERCs (Fig. 2D and Suppl. Fig. 3B). Magnifications and intensity profiles of the antibody signals further demonstrate localization of the E3-ligases within the Z-disc/I-band region or the A-band, respectively (Suppl. Fig. 3B). As many E3-ligases need proteases like calpains for ubiquitination of their substrates we further checked intracellular localization of calpain-1 in cultured ERCs, using immunofluorescence. In the majority of ERCs (> 90%) we found calpain-1 localization in the cytosol and in the I-band of the sarcomere (Suppl. Fig. 3C).

### Downregulation of MuRF1-3 alters titin ubiquitination after autophagy and proteasomal inhibition

**Figure 3:**
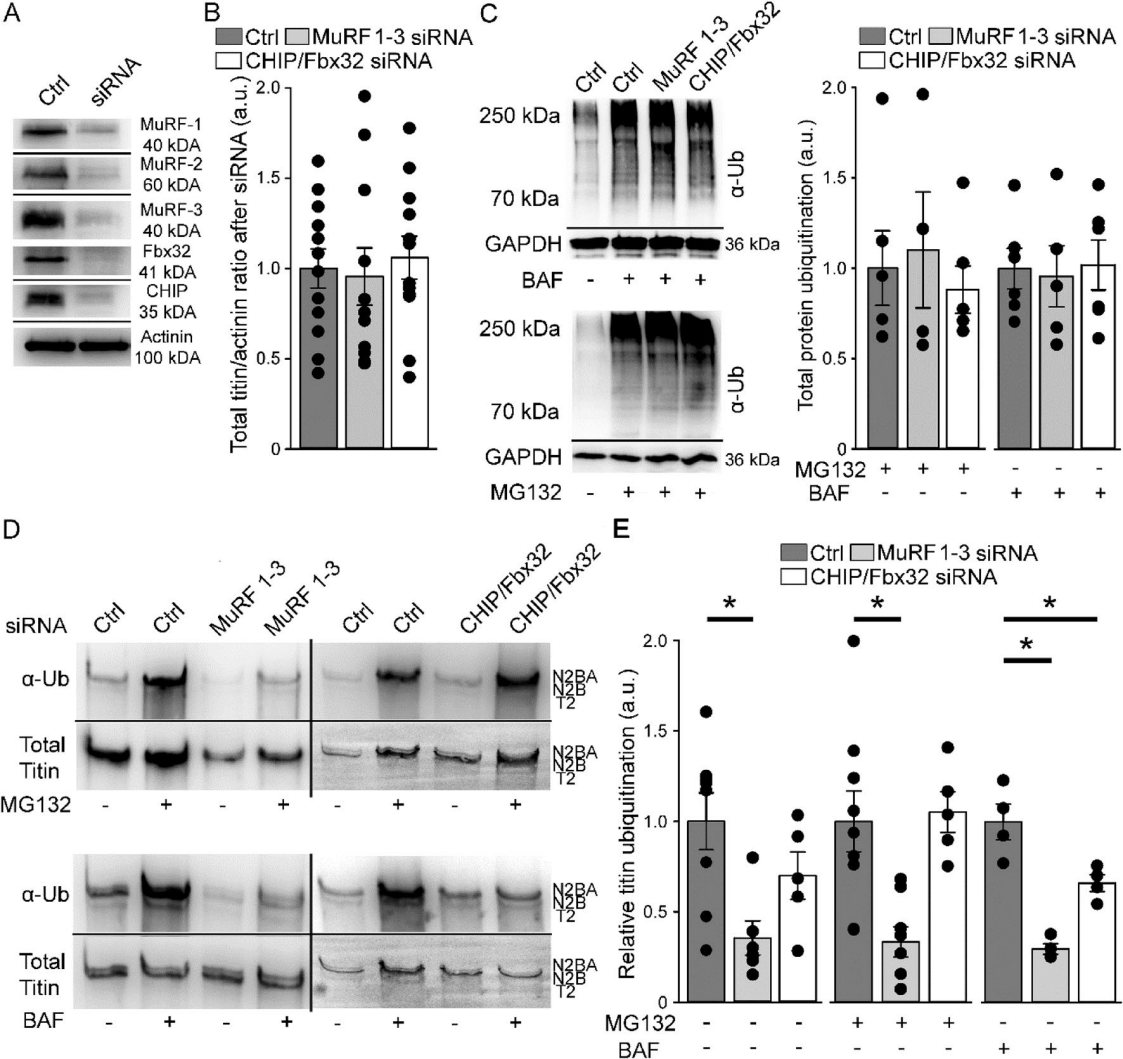
Decreased titin ubiquitination by the knock-down of E3-ligases. Cardiomyocytes were treated with siRNAs and 72 h later with BAF or MG132 for 24 h. (**A**) Representative Western blot images of E3-ligase knock-down. (**B**) Titin/Actinin ratio in siRNA treated cells under basal conditions (n = 11–12). (**C**) Representative Western blot images and analysis of protein ubiquitination in the range of 70–250 kDa in siRNA treated cells after autophagy or proteasomal inhibition (n = 4–6) and. (**D**) Representative Western blot images of siRNA treated cardiomyocytes with or without inhibition. (E) Analysis of titin ubiquitination levels after MuRF-1, -2 and -3 or CHIP/Fbx32 siRNA treatment (n = 5–8). Either Coomassie stained membranes or an antibody targeting titin PEVK was used in titin blot as a marker for titin loading. Data are shown as mean ± SEM. Asterisks (*P* < 0.05 in one-way ANOVA with Bonferroni *t*-test) mark statistical significance. BAF = bafilomycin; Ctrl = control; MG132 = proteasome inhibitor; α-Ub = anti-Ubiquitin antibody.

Due to its gigantic size we hypothesized that titin is likely ubiquitinated by more than just one E3-ligase. We therefore aimed to identify E3 ligases that ubiquitinate the titin filament and treated ERCs at cultivation day 5 for 24 h with siRNAs targeting MuRF-1, -2 and -3, CHIP and Fbx32. After 72 h (cultivation day 8), we additionally inhibited the proteasome or autophagy for 24 h using MG132 and BAF, respectively. If the listed E3 ligases were responsible for proteasome- or autophagy-targeting titin ubiquitination, siRNA-mediated knock-down should prevent increased titin ubiquitination after inhibition by MG132 or BAF. To exclude off target effects of the siRNAs we additionally used two independent siRNA negative controls. Titin ubiquitination after inhibition of autophagy or the proteasome was not altered by the control siRNAs (Suppl. Fig. 4A). Since members of the MuRF family can functionally compensate the loss of each other^18^, we started with knocking down all three MuRFs as well as CHIP and Fbx32 in common to narrow down the candidates. Efficiency of the siRNA treatment was analyzed by Western blot analysis and demonstrated that protein levels of the E3-ligases were markedly decreased by the siRNA treatment (Fig. 3A). Total titin protein levels were not significantly affected by MuRF-1, -2 and -3 or Fbx32/CHIP siRNA treatment without additional inhibition of proteasome or autophagy, but we observed a strong variability in total titin: actinin ratio within groups (Fig. 3B). We hypothesized that at this time of cultivation ERCs have already differentiated to a more adult state. This maturation involves the loss of proliferative ability leading to a substantial reduction in the degradation and synthesis of sarcomeric proteins. In support of this hypothesis we stained for the cell cycle marker Ki67 (Suppl. Fig. 4B) and found an almost complete loss of Ki67 staining after 8 days.

Additional inhibition of autophagy or the proteasome did not reduce ubiquitination of cardiomyocyte protein within the molecular weight range of 70–250 kDa (Fig. 3C). In contrast, levels of ubiquitinated titin were significantly decreased in cells with a knock down of MuRF-1, -2 and -3 under basal conditions (− 64.4% ± 9.43%), after inhibition of autophagy (− 69.3% ± 2.91%) and proteasomal activity (− 66.6% ± 8.37%) (Fig. 3D/E). In cells with a knock-down of CHIP and Fbx32 we determined significantly lower titin ubiquitination levels after inhibition of autophagy (-33.9% ± 4.56%), but not under basal conditions or after inhibition of the proteasome (Fig. 3D/E).

**Figure 4:**
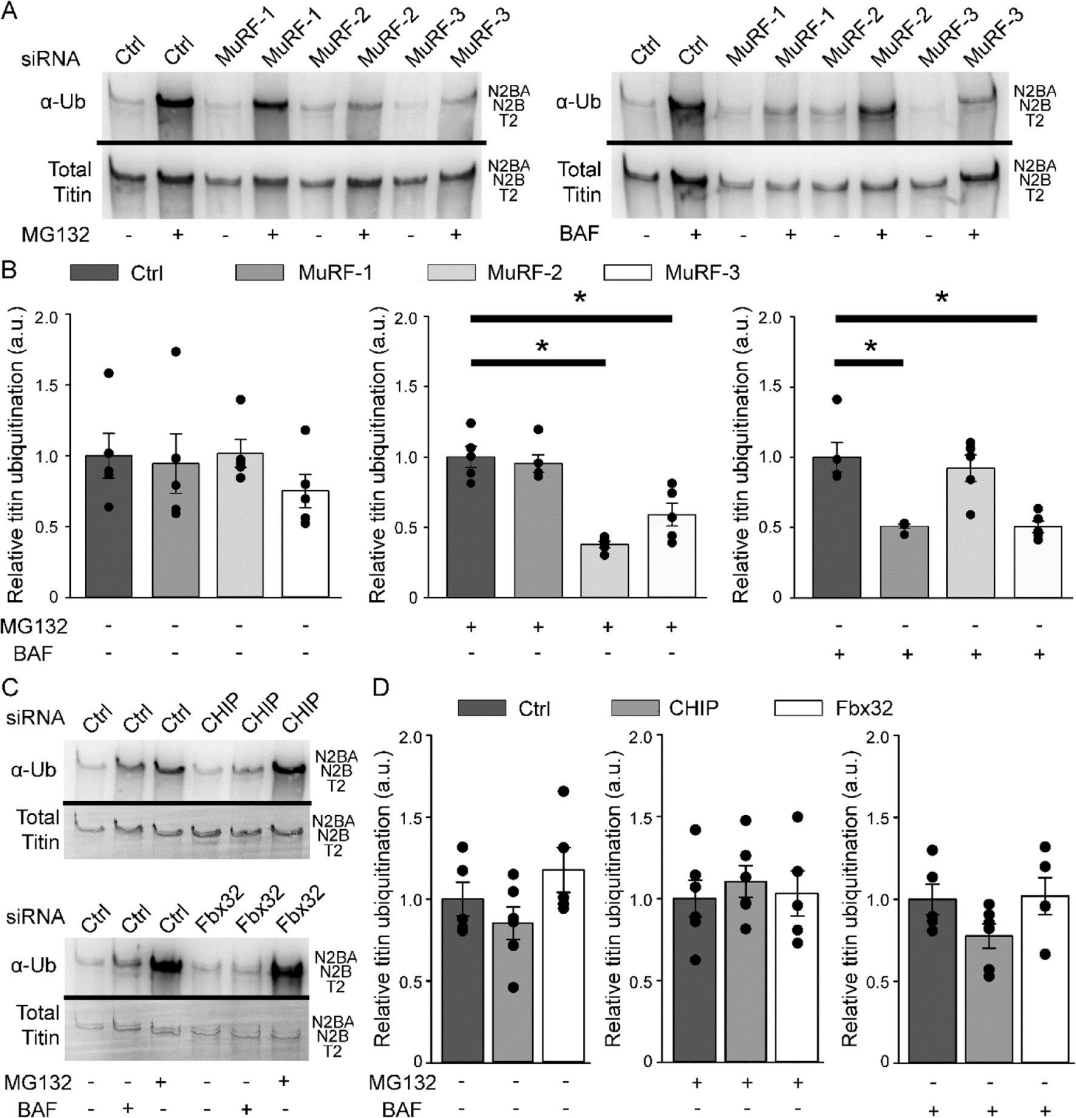
Differential autophagy- or proteasome-related ubiquitination of titin. Cardiomyocytes were treated with siRNAs and 72 h later with MG132 or BAF. (**A**/**C**) Representative Western blot images of siRNA treated cardiomyocytes with or without inhibition. Analysis of titin ubiquitination levels after MuRF-1, -2 or -3 (**B**) or CHIP or Fbx32 (**D**) siRNA treatment. Either Coomassie stained membranes or an antibody targeting titin PEVK was used in titin blot as a marker for titin loading. Data are shown as mean ± SEM (n = 4–6). Asterisks (*P* < 0.05 in one-way ANOVA with Bonferroni *t*-test) mark statistical significance. BAF = bafilomycin; Ctrl = control; MG132 = proteasome inhibitor; α-Ub = anti-Ubiquitin antibody.

To more specifically characterize the E3-ligases we treated cells with single siRNAs targeting either MuRF-1, -2, -3, CHIP or Fbx32 (Fig. 4A/B). In contrast to the knock-down of all three MuRFs single siRNA treatment of MuRF-1, 2 or 3 had no effect on basal titin ubiquitination (Fig. 4B), which underlines the idea of mutual compensation of the MuRF ligases. However, after proteasomal inhibition downregulation of MuRF-2 and -3 significantly reduced titin ubiquitination (− 62.5% ± 2.24% and 41% ± 8.25), whereas after inhibition of autophagy only MuRF-1 and -3 decreased titin ubiquitination (− 49.2% ± 1.51% and − 49.5% ± 4.04%) (Fig. 4B). Of note, single knock down of CHIP and Fbx32 did not affect titin ubiquitination, neither after autophagy nor after proteasomal inhibition (Fig. 4C/D).

## Discussion

In a beating heart, sarcomeres are exposed to permanent mechanical stress. In order to maintain proper cardiac function, turnover and renewal of sarcomeric components as well as adaptation to altered mechanical strain have to be precisely managed by protein quality control mechanisms. In our present study, we present first evidence of K63-dependent poly-ubiquitination of the titin filament, which is strongly increased after inhibition of autophagy. We conclude from these data that degradation of titin is not exclusively performed by the proteasome, but also by autophagy.

To test the efficiency of autolysosomal inhibition the expression level of autophagy marker proteins was tested using immunofluorescence and Western blot analyses. In response to inhibited autophagy, we detected increased protein levels of LC3-II. However, using only LC3-II as marker is not sufficient to draw a conclusion concerning activation or inhibition of autophagy because increased LC3-II protein levels could indicate both higher autophagic activity by elevated LC3-I lipidation and formation of autophagosomes, or impaired lysosomal degradation of unprocessed autophagosomes. In addition, we therefore analyzed p62 protein levels, which binds LC3 at the inner membrane of the autophagosome and becomes degraded after fusion with a lysosome^25^. Protein levels of p62 are increased by inhibition and decreased by activation of autophagy^26^.We also found p62 accumulation after inhibition of the proteasome (Fig. 1B) suggesting that p62 protein levels can increase independent of the formation of autophagosomes. These findings are hints for proteasomal degradation of p62 and its shuttled substrates. Proteasomal inhibition did not affect the protein level of LC3-II (Fig. 1B). The combination of increased LC3-II and p62 expression levels observed in our study can be considered as an indicator for effective autophagy inhibition.

The hypothesis that titin degradation products are in part processed by autophagosomes is further supported by the presence of PEST motifs in the so-called PEVK-domain of the filament protein. Similar to K48-dependent ubiquitination, K63-dependent polyubiquitination preferably occurs at these PEST motifs, which have been described as potent protein degradation–targeting signals^27^. These regions are rich in proline (P), glutamic acid (E), serine (S), and threonine (T). Sequence analysis of titin revealed such PEST motifs in the PEVK domain titin, which is rich in the amino acids proline (P), glutamic acid (E), valine (V) and lysine (K).

Previous reports have demonstrated titin proteolysis by calpains potentially defining calpains as central components of titin turnover^9,28,29^. Matrix-metallo-protease 2 has also been shown to bind within the sarcomere and degrade titin resulting in a higher abundance of the titin degradation product T2 and an increased T2/T1 ratio^21^. It has therefore been widely accepted that a molecule of the size of titin requires pre-digestion for being released from the pseudocrystalline sarcomeric structure. In our present study we report that inhibition of autophagy and proteasomal function resulted in ubiquitination not only of the T2-band but to a large extent of non-processed titin. This finding indicate that ubiquitination occurred at the full-length titin suggesting that the ubiquitination process is independent of and happens before the pre-digest. Nevertheless, calpain and MMP2—mediated proteolysis may be required for E3 ligases to reach some of their target sequences along the titin filament, particularly in the tightly packed A-band region that is closely associated with the myosin filament. The specific titin degradation band T2 was increased after inhibition of autophagy or the proteasome suggesting that under these conditions the titin degradation is stalled after proteolytic pre-digest, resulting in accumulation of the pre-digest product T2. At the same time, protein levels of full-length titin were increased indicating that inhibition of autophagy or the proteasome additionally results in significantly lower amounts of pre-digested full-length titin. This could in part be explained by previous findings reporting that peptide aldehyde inhibitors like MG132 can reduce calpain and MMP2 activity^30,31^. Of note, titin T2 band has a similar molecular weight than the recently identified cronos titin isoform, which is highly expressed early in fetal cardiomyocyte development and has been suggested to be important for normal sarcomere maturation and function^32^. We currently cannot exclude the possibility that in the ERC experiments part of the observed increase in the T2 band may derive from higher expression of the titin cronos isoform.

**Figure 5:**
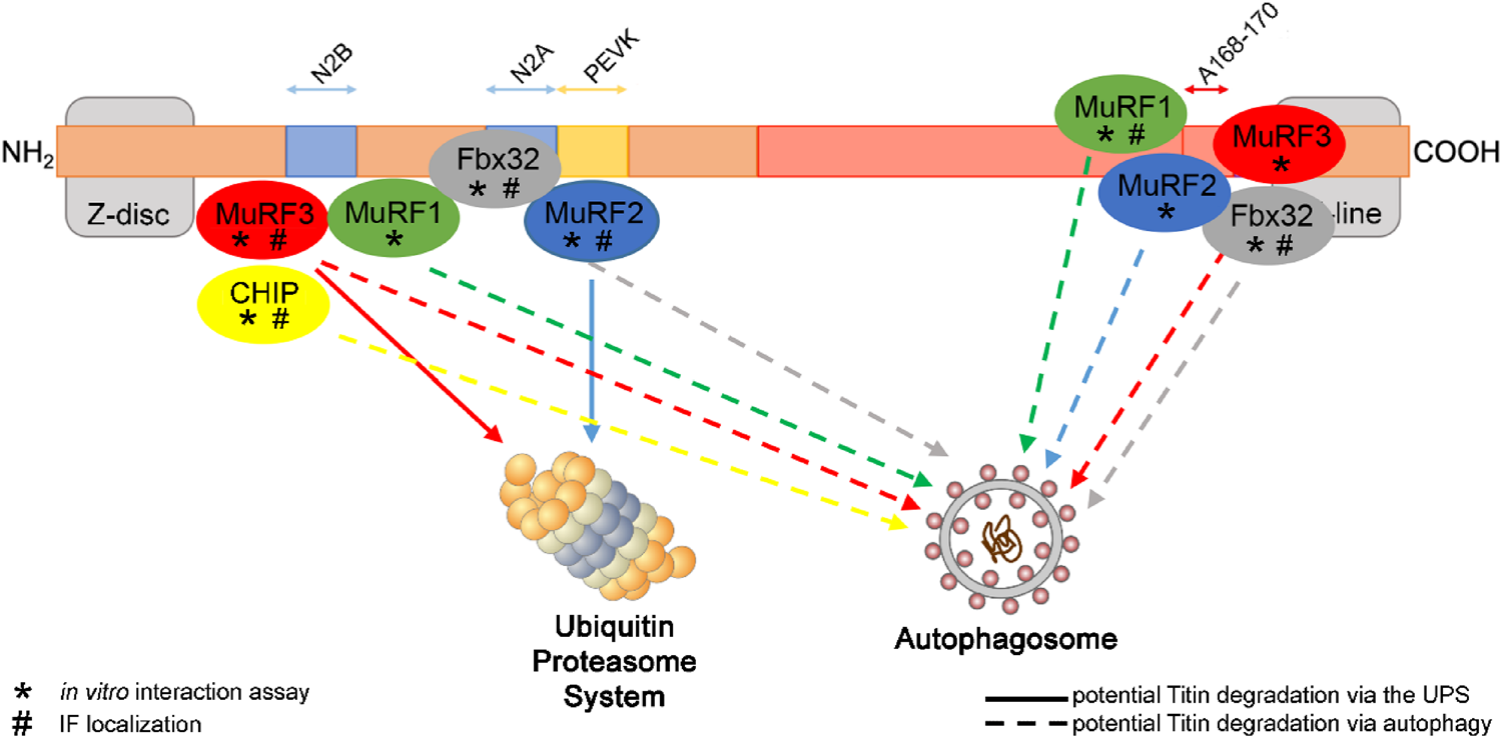
Schematic view of E3-ligase interaction and modification of the titin filament. The scheme illustrates a possible regulation of E3-ligase based titin ubiquitination and subsequent degradation by autophagy or the proteasome based on the in vitro pull down experiments and immunofluorescence data. Straight lines represent potential degradation via the proteasome and dashed lines degradation via autophagy after ubiquitination by the respective E3-ligase. NH2 = titin N-terminus; COOH = titin C-terminus; * = identified by in vitro interaction assay; # = identified by immunofluorescence stainings.

We further aimed to identify E3-ligases involved in titin degradation via the proteasome and autophagy and performed in vitro pull-down experiments with selected E3 ligases and recombinant titin fragments. Immunfluorescent stainings of E3 ligases in ERC and ARC confirmed the observations from pull down experiments: MuRF-1 was detected within the A-band/M-line and showed binding to the titin fragment A168-170. We also observed binding of MuRF-1 to the titin N2B-domain, which may provide an explanation for the previous observation that MuRF-1 localizes at the Z-disc/I-band region^33^. We found MuRF-2 localization in the Z-disc/I-band region of cultured embryonic and adult rat cardiomyocytes, which is line with its in vitro binding to the titin PEVK-domain. Unlike Lange et al.^22^ we did not observe MuRF-2 localization within the A-band/M-line. We further identified MuRF-3, Fbx32 and CHIP as novel interaction partners of titin. Our data suggest interaction of MuRF-3 and Fbx32 with the titin A168/170 domain, which is supported by their sarcomeric localization at the M-line of cultured cardiomyocytes. Fbx32 further showed binding affinity to the N2B and N2A titin fragments and intracellular localization in Z-disk proximity in adult cardiomyocytes. This is in line with a previous report showing localization of Fbx32 at the Z-disk^28^. Of note, in the fluorescence microscope used here, it was not possible to clearly distinguish between Z-disk and I-band localization. Therefore, a signal in Z-disk proximity is quite plausible for Fbx32 binding to the N2A or N2B segment. Considering the gigantic size of titin, binding of E3-ligases to other titin domains than the ones tested here is of course possible. Also unspecific or non-functional binding of E3-ligases to titin in the immunofluorescence stainings cannot be entirely excluded. Figure 5 summarizes the main findings of the interaction and localization studies and illustrates possible regulation of E3-ligase based titin ubiquitination and degradation by autophagy or the proteasome.

We next aimed to establish the functional importance of the identified E3 ligases by targeted deletion using specific siRNAs and determined the effects on titin ubiquitination. Ubiquitination of titin has previously been shown in skeletal muscle by Western blot analysis and electron microscopic images using ubiquitin antibodies^18^. However, the study did not determine whether titin was targeted for degradation by autophagy or the proteasome. Here we provide direct evidence for ubiquitination of titin by five E3-ligases. In addition, our study discriminates for the first time between autophagy and proteasome dependent ubiquitination of titin. The members of the MuRF family are able to build hetero-dimers and thereby target different domains within the titin filament for ubiquitination. Varying hetero-dimers could also explain autophagy- or proteasome-related ubiquitination. Other reports assumed a redundancy of MuRF members concerning degradation of several target proteins^18^. Here, knocking down single MuRFs in cardiomyocyte cultures differentially altered titin ubiquitination, indicating that MuRF 1, -2 or -3 are not able to compensate the loss of each other completely. Simultaneous knock-down of CHIP and Fbx32 also significantly lowered titin ubiquitination after autophagy inhibition. Why single knock down of Fbx32 and CHIP in our model did not significantly alter titin ubiquitination still remains elusive at this point. CHIP has been reported to play an important role in ischemia–reperfusion injury as knock out of CHIP worsens the outcome in mouse models^19^. Our group previously demonstrated that titin ubiquitination is strongly increased in the non-ischemic area during the first days after cardiac ischemia, possibly because of increased turnover in response to elevated mechanical stress^23^. We now hypothesize that this titin ubiquitination is in part performed by CHIP and the other E3-ligases described here. Decreased titin ubiquitination and impaired titin degradation could therefore contribute to the detrimental effects of CHIP knockdown for myocardial performance after ischemia^19^.

Somewhat unexpectedly, downregulation of E3-ligases did not result in differences in total titin protein levels compared to untreated cells, suggesting that titin degradation was not altered by the siRNA treatment. Another unexpected result was the unchanged protein ubiquitination in the molecular weight section from 70 to 250 kDa after siRNA treatment and inhibition of autophagy or the proteasome. A possible explanation for both observations could be the fact that E3-ligases were strongly reduced by the siRNA treatment, but not completely absent so sufficient levels of ubiquitination and turnover may still occur. Besides, it is conceivable that MuRFs can compensate for the loss of CHIP and Fbx32 and vice versa. Other cardiac E3-ligases like Casitas b-lineage lymphoma (c-Cb1), ubiquitin-protein ligase E3A (UBE3A/E6AP), cellular inhibitor of apoptosis (cIAP) or F-box and leucine-rich repeat protein 22 (Fbxl22)^34^ may also compensate for the loss of others.

The binding assays showed two main clusters of E3-ligase interaction with titin: one in the I-band region, the other at the A-band/M-band junction (summarized in Fig. 5). The Titin T2 fragment, as a result of the pre-digest, has been reported to contain mainly the A-band part of titin^6^. From our data we conclude that particularly MuRF2 and MuRF3, which bind to titin in the I-band region, could be responsible for I-band titin ubiquitination and its subsequent processing by the UPS. The A-band part of titin is tightly associated to the myosin-filaments and therefore not easily accessible, even after proteolytic removal of the proximal I-band part. We therefore hypothesize that the entire A-band part of titin could be degraded via autophagy. Along the same line, p62, which associates to the titin kinase domain in close M-line proximity^22^ possibly serves as an anchor protein for the formation of autophagosomes.

Unfolded domain oxidation (UnDOx) within the titin filament was recently reported to alter titin-based properties^35^. Many pathologies including ischemia are linked to alterations of the oxidative state. Titin oxidation has been shown to occur in the ischemic region of hearts from mice that underwent permanent ligature of the LAD^35^. Unfolding events may also increase the vulnerability of the titin filament and subsequently ubiquitination and degradation.

Revealing the mechanisms involved in titin turnover is important also from a clinical point of view, as disturbances of protein degradation are hallmarks of several diseases including cardiovascular diseases^36^. Insufficient titin turnover and modification of myocardial elasticity could be a part of this dysregulation. In skeletal muscle M-line titin has been reported to be a binding partner as well as a substrate for calpain-3 and impaired titin-calpain interaction causes titinopathies with increased titin turnover^9,29^. Titin-truncating variants are the most common genetic cause of dilated cardiomyopathy^37^, which is often associated with changes in the cellular metabolism and decreased autophagic degradation of myocardial proteins. In turn, impairment of autophagy or the proteasome could lead to accumulation of dysfunctional titin filaments or even titin aggregates. Pathological hyper-activation of the degradation systems could potentially result in mislead turnover of functional filaments. Both situations likely affect passive stiffness and diastolic properties of the myocardium.

## Conclusion

Taken together, we demonstrated that protein quality control of the sarcomeric titin filament not only involves degradation by the proteasome but also by the autolysosomal system. Our data further suggest that ubiquitination of full-length titin does not necessarily require proteolytic pre-digestion. Finally, we propose that different muscle specific E3-ligases specifically ubiquitinate titin domains for targeted degradation by either autophagy or the proteasome. We conclude that more detailed knowledge of the fine-tuned ubiquitination and turnover process of titin is needed to understand how pathological modification of these processes may influence sarcomere function and affect the properties of the myocardium.

## Materials and methods

### Isolation of embryonic rat cardiomyocytes (E18)

All animal procedures were conducted in accordance with the local animal care and use committee and the responsible authorities (Landesamt für Natur, Umwelt- und Verbraucherschutz Nordrhein-Westfalen) that reviewed and approved the experimental protocols (Az. 84–02.04.2017.A145). Hearts were obtained from embryos (gestational day 18) of pregnant adult Wistar rats (Janvier Labs). Adult animals were anesthetized with ketamine/xylazin (100 and 10 mg/kg body weight) embryos were removed by C-section and embryos as well as the adult animal were euthanized by decapitation. Embryonic rat cardiomyocytes (ERC) were isolated by enzymatic digestion of the embryonic heart and plated in 6-well dishes (day 0). Cells were cultured in DMEM + 20% FBS (culture medium) or serum-starved (DMEM + 1% charcoal filtered serum). ERCs were plated on gelatine-coated (1% in PBS) cover slips. Cells were then fixed with 2% paraformaldehyde. Primary antibodies were incubated overnight at 4 °C. Rabbit or mouse AlexaFluor488 and AlexaFluor555-coupled secondary antibodies were incubated for 2 h at room temperature. For inhibition of autophagy or proteasomal activity medium was supplemented with chloroquine (CQ, 10 µM, Sigma), bafilomycin (BAF, 100 nM, Cayman Chemical Company) or MG132 (1 µM, Sigma) for up to 24 h. The study was carried out in compliance with the ARRIVE guidelines.

### SiRNA treatment in embryonic rat cardiomyocytes (E18)

Rat specific siRNAs (Dharmacon) were used for downregulation of E3-ligases MuRF-1, MuRF-2, MuRF-3, CHIP and Fbx32 in embryonic rat cardiomyocytes following the manufacturers siRNA transfection protocol. DharmaFECT™ 2 transfection reagent (Dharmacon) was used for all experiments. At cultivation day 5 serum- and antibiotic-free DMEM medium was supplemented with a final siRNA concentration of 25 nM and added to the cells for 24 h. For control cells, only the transfection reagent was added. To exclude the possibility for off target effects we additionally used two independent siRNA negative controls (MISSION siRNA Universal Negative Control #1 and #2; Sigma). At cultivation day 6 medium was changed back to culture medium and cells were cultivated for additional 48 h. Finally, cells were treated with BAF or MG132 for 24 h and harvested for biochemical analysis. Effective downregulation of the target proteins was analyzed by Western blot.

### Isolation of adult rat cardiomyocytes

Adult rat CMs were isolated using a Langendorff perfusion system, as previously described^38,39^. Briefly, male Wistar rats were terminally anaesthetized with isoflurane and killed by cervical dislocation. The heart was dissected and the aorta was cannulated and connected to a perfusion apparatus. After perfusion with gassed digestion buffer single cardiomyocytes were isolated (for detailed information see online supplement). For immunofluorescent stainings cells were plated on laminin/gelatine coated cover glasses. Cells were then fixed with 2% paraformaldehyde. Primary antibodies were incubated overnight at 4 °C. Rabbit or mouse AlexaFluor488 and AlexaFluor555-coupled secondary antibodies were incubated for 2 h at room temperature.

### Sodium dodecyl sulfate–polyacrylamide gelelectrophoresis (SDS-PAGE) and Western blot analysis

For protein analyses samples were solubilized in modified Laemmli buffer, titin isoforms were separated on agarose-strengthened 2.2% polyacrylamide gels, other proteins were separated using standard 7.5–15% polyacrylamide gels^23^. Proteins were visualized by imperial protein staining solution (Thermo Scientific). For analyses gels were scanned using a Fusion FX imager (Vilber & Lourmat, France) and analyzed densitometrically (Image J). Titin degradation was measured by calculating the ratio of the typical titin degradation product T2 and full-length total titin (N2BA + N2B = T1). Results were normalized to control levels and shown as relative T2/T1 ratio. Alterations of total titin after inhibition of autophagy or the proteasome were determined by the titin/actinin ratio. For Western blot analysis, proteins were transferred to PVDF membrane and protein transfer was controlled by reversible Coomassie stain. Membranes were cropped to the estimated molecular weight range before blocking and incubation with the respective antibodies to detect several proteins with different molecular weights simultaneously. After blocking in TBST + 2% BSA membranes were incubated with the following primary antibodies: α-LC3 (Cell signaling, #2775), α-SQSTM1/p62 (Cell signaling, #5114 or Abcam, ab56416), α-ubiquitin (Cell signaling, #3936), α-K63-polyubiquitin (Cell signalling, #5621), α-Fbx32 (Abcam, ab74023 or ECM, AP2041), α-MuRF-1 (Abcam, ab172479), α-MuRF-2 (Abcam, ab4387), α-MuRF-3 (Acris, ARP43486), α-Stub1/CHIP (Cell Signaling, #2080), α-Titin-PEVK (Eurogentec), α-actinin2 (Sigma, A7811/A7732) and α-GAPDH (Sigma Aldrich, G8795). Secondary antibodies were fused with horseradish-peroxidase. Bands were visualized using a Fusion FX imaging system and signal intensity was analyzed densitometrically (Image J).

### Human heart tissues

Left ventricular human heart tissue was recovered from non-failing donor human hearts rejected for transplantation from the Mid-American Transplant Service (St. Louis, MO) and kindly provided by Igor Efimov (see acknowledgement). Upon release of the organ for research purposes hearts were obtained immediately after removal from the chest in the operating room arrested in ice-cold cardioplegic solution, and transported to the laboratory for tissue preparation. Heart tissue was kept at − 80 °C until further use. All studies using human heart tissue have been approved by the Institutional Review Board at Washington University in St. Louis.

### Messenger RNA isolation and cDNA synthesis

Messenger RNA was isolated from human left ventricular tissues using RNeasy Fibrous Tissue Kit (Qiagen), according to manufacturer protocol and quantified by Nanodrop™ 2000c UV–Vis Spectrophotometer (Thermo Fisher Scientific). 200 ng of the human RNA was reverse transcribed into cDNA with random primers using Quantitect Reverse Transcription Kit (Qiagen) to generate prokaryotic expression constructs for different human titin fragments and human E3-ligases.

### Cloning and protein purification

Prokaryotic expression constructs for different titin fragments and E3-ligases were produced for expression in *E. coli* and subsequent affinity purification. DNA fragments were obtained by PCR using human cDNA. PCR products were ligated into pGEX-4 T-1 expression vector. The generated constructs for titin fragments according to NCBI accession numbers nm_003319 (N2B isoform), nm_133378 (N2A isoform) and nm_001256850.1 (N2BA-Isoform) were as follows: N2-B region (exon 49), N2A (exons 102–109), a constitutively expressed PEVK segment (exons 219–225) and A168-170 (Ig141/Ig142/FN3-132). Human E3 ubiquitin ligases MuRF-1 (= TRIM63, nm_032588.4), MuRF-2 (= TRIM55, nm_184085.2), MuRF-3 (= TRIM54, nm_187841.3), CHIP (= Stub1, nm_005861.4) and Fbx32 (nm_148177) were cloned in pGEX-4 T-1 vector as well. All constructs were verified by Sanger-sequencing. Vectors were transformed in competent *E. coli* NEB Express (New England Biolabs) and expression was induced using 0,2 mM isopropyl-ß-d-thiogalactopyranoside. Fragments were purified using glutathione affinity chromatography. Thrombin (10–20 U) cleavage separated the titin fragments from their GST-tag. Para-aminobenzamidine sepharose beads were used to eliminate thrombin from the solution.

### Pull down interaction assays

All human E3-ligases and titin fragments were recombinant expressed and purified with the GST-Fusion System. With the GST-tag E3-ligases were immobilized on GSH-sepharose beads and incubated with supernatant containing purified titin fragments for 90 min at 4 °C. All titin fragments were tested for an affinity to the GST-tag. The beads were washed four times to eliminate unbound protein, and the interaction was detected by Western blotting with overnight incubations of anti-titin antibodies (α-PEVK; α-N2B; α-N2A and α-A186) and 1 h incubations of horseradish peroxidase–conjugated anti-rabbit secondary antibody (dilution 1:10.000). Bands were visualized using a Fusion FX imaging system (Vilber & Lourmat, France). Binding affinity was analyzed by calculating the ratio of input signals to bound signals.

### Statistical analysis

Data are presented as mean ± standard error of the mean (SEM). Statistical significance was analyzed by using unpaired Student *t* test. Multiple comparisons were performed using one-way ANOVA Bonferroni *t*-test or Dunn`s multiple comparison. *P* values < 0.05 were considered significant and are indicated in the figures by asterisks. Analysis was performed using Sigma Plot (version 13.0).

## Supplementary Information

Supplementary Information.

## Data Availability

Raw data and supporting data and methods will be made available to editors and reviewers upon request.

## Abbreviations

ARC: Adult rat cardiomyocytes
BAF: Bafilomycin
CHIP: Carboxy terminus of Hsp70-interacting protein
CQ: Chloroquine
DMEM: Dulbeccos modified eagle medium
ERC: Embryonic rat cardiomyocytes
FBS: Fetal bovine serum
GAPDH: Glycerinaldehyde-3-phosphate-dehydrogenase
MMP2: Matrix metallo protease 2
mTOR: Mammalian target of rapamycin
MuRF: Muscle specific ring finger
N2B/N2BA: Cardiac titin isoforms
N2A: Skeletal muscle titin isoform
PEVK: Proline, glutamate, valine and lysine rich domain of titin
PFA: Paraformaldehyde
PQC: Protein Quality Control
SL: Sarcomere length
T2: Specific titin degradation band
TRIM: Tripartite motif
UPS: Ubiquitin proteasome system
