# Supplementary material for: E3-ligase knock down revealed differential titin degradation by autophagy and the ubiquitin proteasome system
Source: Sci Rep. 2021 Oct 26;11:21134. doi: 10.1038/s41598-021-00618-7 (PMC8548520; doi:10.1038/s41598-021-00618-7)
Supplement: Supplementary file 1 — Supplementary Information. [file 41598_2021_618_MOESM1_ESM.pdf]

Cardiac Titin ubiquitination and degradation by autophagy and the ubiquitin-proteasome-system

Erik Müller[1], Senem Salcan[1], Sabine Bongardt[1], David Monteiro Barbosa[1], Martina Krüger[1], Sebastian Kötter[1#]

[1]Department of Cardiovascular Physiology, Medical Faculty and University Hospital Düsseldorf, Heinrich-Heine-University Düsseldorf, Germany

#Correspondence to:
Dr. Sebastian Kötter
Dept. of Cardiovascular Physiology
Medical Faculty
Heinrich Heine-University Düsseldorf
Universitätsstr. 1, 22.03 02
D- 40225 Düsseldorf, Germany
Phone: +49-(0)211-81-10662
Fax: +49-(0)211-81-12672
sebastian.koetter@uni-duesseldorf.de

Supplement

Materials and Methods

Sodium dodecyl sulfate-polyacrylamide gelelectrophoresis and Western blot analysis

For titin isoform composition analysis and standard sodium dodecyl sulfate polyacrylamide-gelelectrophoresis (SDS-PAGE) samples were solubilized in modified Laemmli buffer [1] and proteins separated on agarose strengthened 2.1-2.3 % PA titin gels stabilized with agarose, or 7.5-15 % PA standard gels. Proteins were visualized by imperial protein staining solution (Thermo Scientific). For densitometric analysis gels were scanned using a Fusion FX imager (Vilbert & Lourmat) and analyzed densitometrically (Image J). Average titin-isoform composition was calculated from a minimum of n = 3 repetitions per experiment. Titin degradation was measured by calculating the ratio of full-length total titin (N2BA + N2B) and the typical titin degradation product T2. Results were normalized to control levels and shown as relative T2/T1 ratio. Alterations of total titin after inhibition of autophagy, the proteasome or siRNA treatment was determined by the titin/actinin ratio. For Western blot analysis proteins were transferred onto PVDF membrane and the protein transfer was controlled by reversible Coomassie stain. Membranes were cropped to the estimated molecular weight area before blocking and incubation with the respective antibodies. After blocking in TBST + 2% BSA membranes were incubated with primary antibodies: α-LC3 (Cell signaling, #2775), α-SQSTM1/p62 (Cell signaling, #5114 or Abcam, ab56416), α-ubiquitin (Cell signaling, #3936), α-K63-polyubiquitin (Cell signalling, #5621), α-Fbx32 (Abcam, ab74023 or ECM, AP2041), α-MuRF-1 (Abcam, ab172479), α-MuRF-2 (Abcam, ab4387), α-MuRF-3 (Acris, ARP43486), α-Stub1/CHIP (Cell Signaling, #2080), α-titin-PEVK (Eurogentec or Immunoglobe), α-actinin 2 (Sigma, A7811/A7732) and α-GAPDH (Sigma Aldrich, G8795). Secondary, horseradish-peroxidase conjugated anti-rabbit (Cell Signaling, #7074) or anti-mouse (Cell Signaling, #7076) antibodies were used. All membranes were stripped between the ubiqitin-specific and the titin-PEVK antibody. Briefly, membranes were incubated with stripping buffer (6M GnHCl, 0.2% Nonidet P-40 (NP-40), 0.1M β-mercaptoethanol, 20mM Tris-HCl, pH7.5) for 30 minutes to remove the primary and secondary antibodies. The blots were then washed several times with TBST to remove β-mercaptoethanol. Stripped membranes probed with secondary antibody did not show protein bands, thus confirming complete removal of the primary antibodies. Importantly, the blotted protein was not removed by this procedure. Bands were visualized using a Fusion FX imaging system (Vilber & Lourmat, France) and signal intensity was analyzed densitometrically (Image J).

Preparation of embryonic (E18) and adult rat cardiomyocytes

All animal procedures were conducted in accordance with the animal care and use committee of the responsible authorities (Landesamt für Natur, Umwelt- und Verbraucherschutz Nordrhein-Westfalen) that reviewed and approved the experimental protocols (Az. 84-02.04.2017.A145). Hearts were obtained from embryos (gestational day 18) of pregnant adult Wistar rats. Animals were anesthetized with ketamine/xylazin, embryos were removed by C-section and embryos as well as the adult animal were euthanized by decapitation. Hearts were minced and cardiomyocytes were isolated by enzymatic dissociation with collagenase Type II (Gibco,1 mg/ml) and trypsin (Gibco, 3 mg/ml) in dissociation buffer (137 mM NaCl, 11 mM D-Glucose, 2.7 mM KCl, 12 mM $NaHCO_3$, 417 µM $NaH_2PO_4 \cdot H_2O$, 56 µM phenol red). Enzymatic dissociation was performed at 37°C for 30-45 min. Additional mechanical dissociation was done by repeated pipetting of the solution. After the dissociation procedure the solution was filtered through a 70µm cell strainer (BD Falcon) and the enzymatic reaction was stopped by adding ten volumes of culture standard DMEM medium (+ 20% fetal bovine serum (FBS), 1% non-essential amino acids, 1% Pen/Strep, 50 µM β-mercaptoethanol). Cells were pelleted by

5 min centrifugation at 1000 rpm, resuspended in standard culture medium, plated at a density of 5x 10^5 cells/well and cultured at 37°C and 5% CO_2. On the next day the medium was changed and cells were treated with mitomycin (30 µM; Sigma M4287) for 1 hour to prevent further proliferation of fibroblasts. Afterwards the medium was changed to standard DMEM medium or low FBS culture medium (DMEM+1% charcoal-filtered FBS). Chloroquine (CQ, 10µM, Sigma), bafilomycin (BAF, 100 nM, Cayman Chemical Company) or MG132 (1µM, Sigma) were applied for up to 24 hours. Cardiomyocytes used for biochemical experiments were harvested in modified Laemmli sample buffer and directly used for SDS-PAGE. For immunofluorescence stainings ERCs were plated on gelatine (1% in PBS) coated cover slips. At culture day 1 the cells were serum-starved (1% charcoal-filtered FBS) and cultured for 3 or 8 days. Cells were then fixed with 4% paraformaldehyde (PFA) for 20 minutes. Cover slips were washed with PBS followed by 5 min Triton treatment (2% Triton X-100 in PBS+2% BSA). Incubation with α-SQSMTS/p62 (Abcam, ab56416), α-LC3 (Cell Signaling; #2775), α-calpain-1 (Acris, 10538-1-AP), α-Fbx32 (Abcam, ab74023 or ECM, AP2041), α-MuRF-1 (Abcam, ab172479), α-MuRF-2 (Abcam, ab4387), α-MuRF-3 (Acris, ARP43486), α-Stub1 (Cell Signaling, #2080) and α-Ki67 (Abcam, ab16667) was performed overnight at 4°C in PBS + 2% BSA. For Identification of cardiomyocytes α-actinin 2 (Sigma-Aldrich, A7811/A7732) or α-titin PEVK (Eurogentec or Immunoglobe) have been used, followed by staining with Alexa488-(Cell Signaling, α-mouse CS4408, α-rabbit CS4412) or Alexa555-conjugated (Cell Signaling, α-mouse CS4409, α-rabbit CS4431) secondary antibodies (1:500) for 1 h at RT. After washing the coverslips were mounted in ProLong Gold antifade reagent with DAPI.

Adult rat CMs (ARC) were isolated using a Langendorff perfusion system, as previously described [2;3]. Adult male Wistar rat with an age of 8 to 12 weeks were terminally anesthetized with isoflurane and killed by cervical dislocation. The thorax was then opened and the heart was injected with heparin solution (78 U/ml heparin) to avoid coagulation and allow proper perfusion and then was removed by dissecting the aorta. Afterwards the heart was placed in a petri dish filled with cold heparin solution and weighed to calculate the appropriate perfusion time. The aorta was then cannulated and connected to a perfusion apparatus filled with perfusion buffer + heparin (130 mM NaCl, 5.4 mM KCl, 1.25 mM NaH_2PO_4, 25 mM HEPES, 20 mM glucose, 3 mM pyruvate, 5 mM creatine, 2 mM carnitine, 5 mM taurine, pH 7.3-7.4; heparin 11 U/ml). The heart was perfused with perfusion buffer at a flow rate of 7 mL/min. After approx. 5 minutes the perfusion buffer was switched to digestion buffer containing collagenase type II (85 U/ml, Worthington), protease from *Streptomyces griseus* (6 U/ml, Sigma) and $CaCl_2$ (50 nM) aerated with 100% oxygen and the heart was perfused for 25-35 min. The heart was carefully removed from the perfusion apparatus and transferred into digestion buffer (100 nM $CaCl_2$, 1.5 mM BSA). Here, the heart tissue was smoothly minced, transferred into a 50 mL falcon and incubated in a water bath for 15 minutes at 37 °C. The solution was mixed by pipetting every 5 min. Thereafter, the solution was filtered through a 200 µm filter to remove remaining non-dissolved tissue. The cells were pelleted by centrifugation for 1 minute at 500 rpm and the supernatant was discarded. The cells were resuspended in low-calcium solution (0.2 mM $CaCl_2$, 1.5 mM BSA) and centrifuged as described above. This step was repeated with a high-calcium solution (0.4 mM $CaCl_2$, 1.5 mM BSA). The pellet was then resuspended and transferred into pre-warmed M199 Hanks cell culture medium (10 % FBS superior, 1 % Penicillin/Streptomycin, 5 mM creatine, 2 mM carnitine, 5 mM taurine, 10 mM HEPES). Cells were left for about 10 minutes at 37°C. Thereafter the cells were resuspended in M199 Hanks cell culture medium supplemented with blebbistatin (8.5 nM) to prevent contraction of the cells. For immunofluorescence stainings ARCs were plated on gelatine/laminin (1% in PBS) coated cover slips. Fixation and staining procedure was the same as described for embryonic rat cardiomyocytes.

Pull down interaction assays

In vitro binding assays were performed using recombinant fragments of human titin and human E3 ubiquitin ligases. Proteins were recombinant expressed in E. coli K12 and purified by the GST-Fusion System. E3-ligases were immobilized on GSH-sepharose beads via the GST-tag and incubated for 90 minutes with supernatant containing purified titin fragments at 4°C. Afterwards beads were washed four times to eliminate unspecific binding and unbound protein. Samples were loaded on 10-12.5% SDS-PAGE and blotted on PVDF membranes followed by overnight incubations of the respective anti-titin antibodies and 1h incubations of horseradish peroxidase–conjugated anti-rabbit secondary antibody (dilution 1:10.000). The last wash step was also loaded on the gels (last wash) to demonstrate that all non-bound protein was washed out. Signals in the *Bound* lanes are tantamount with binding of the E3-ligase and the titin fragment. Bands were visualized using a Fusion FX imaging system (Vilber & Lourmat, France). Binding affinities were analyzed by the ratio of input vs bound signal.

Supplemental references

1.) Kötter S., et al. Titin-Based Cardiac Myocyte Stiffening Contributes to Early Adaptive Ventricular Remodeling After Myocardial Infarction. *Circ Res.*;***119***(9), 1017-1029 (2016). doi:https://doi.org/10.1161/CIRCRESAHA.116.309685

2.) Müller A. E., et al. Acute exercise modifies titin phosphorylation and increases cardiac myofilament stiffness. *Front. Physiol.*;***5***, 449 (2014). doi:https://doi.org/10.3389/fphys.2014.00449

3.) Skrzypiec-Spring M., Grotthus B., Szelag A.Schulz R. Isolated heart perfusion according to Langendorff---still viable in the new millennium. *J. Pharmacol. Toxicol. Methods*;***55***(2), 113-126 (2007). doi:10.1016/j.vascn.2006.05.006

Supplemental figure 1

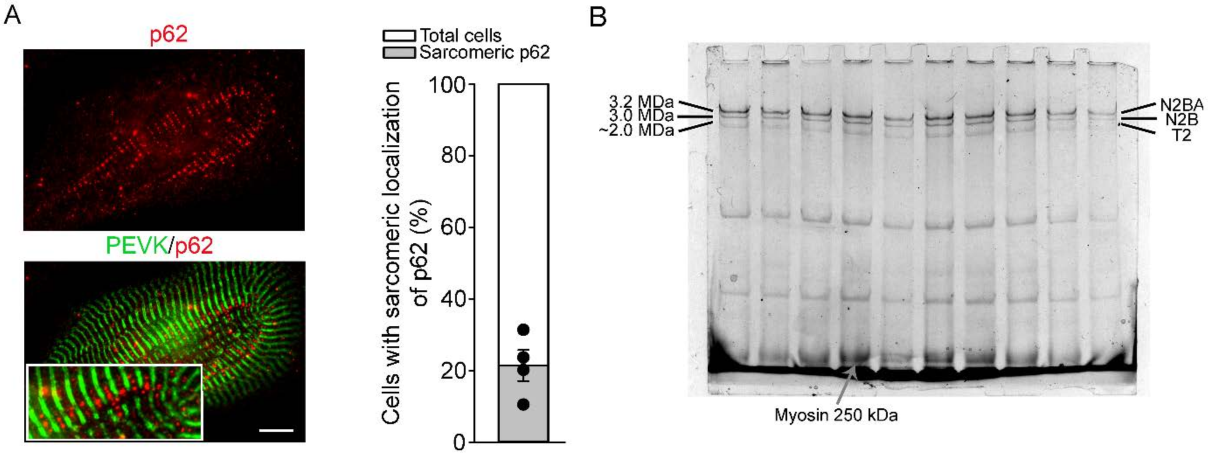

Supplemental fig. 1: Sarcomeric localization of p62 and representative titin SDS-PAGE. (A) Immunofluorescence stainings of p62 (red) in embryonic rat cardiomyocytes. Titin-PEVK antibody was used as a sarcomeric marker. Bar graph shows the percentage of cells with sarcomeric p62 localization (n = 215 cells from 4 different cell preparations). **(B)** Representative titin 2.2 % SDS-PAGE to demonstrate that no other proteins occur in the molecular weight range of titin. Data are shown as mean ± SEM. N2B and N2BA = cardiac titin isoforms; T2 = specific titin degradation product. Bar = 10µm.

Supplemental figure 2

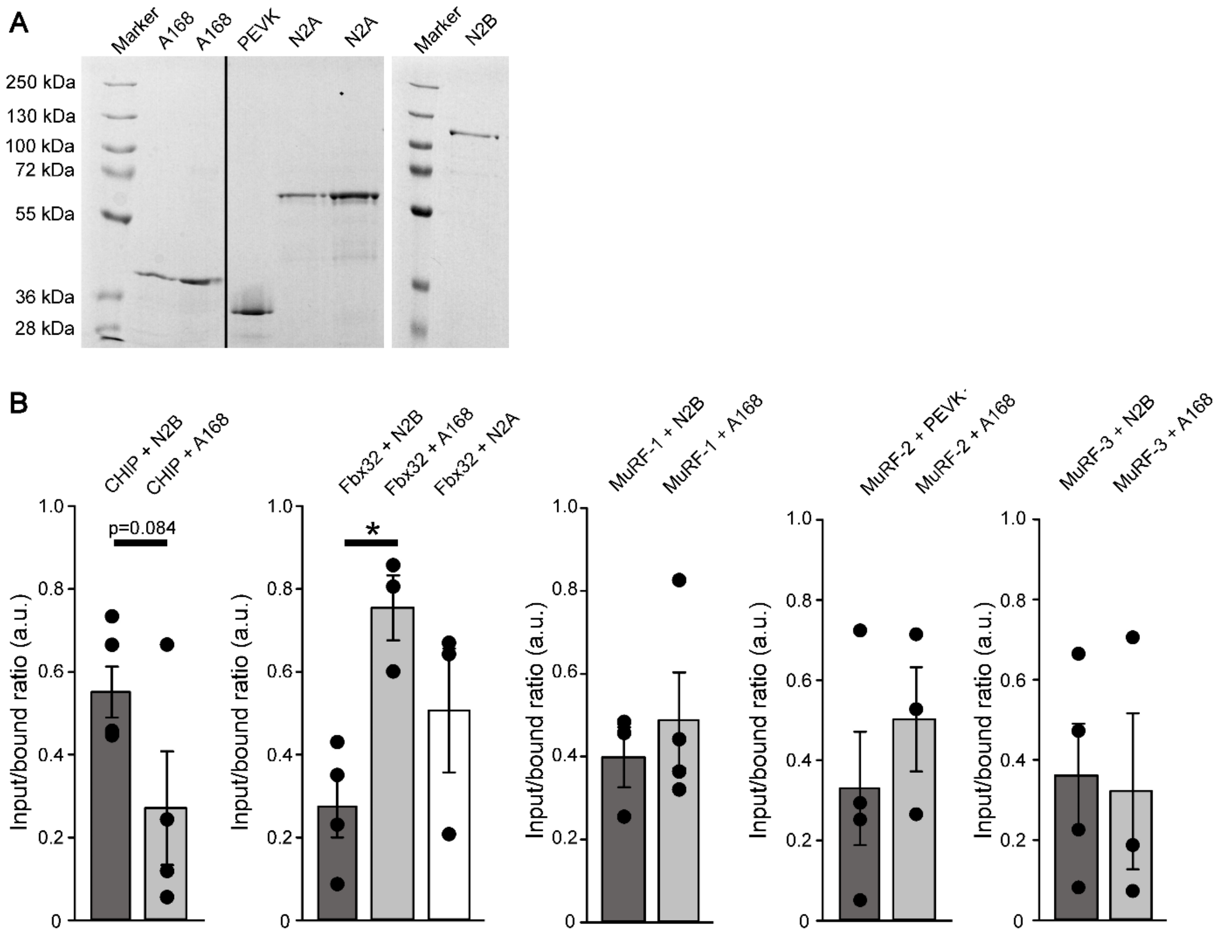

Supplemental figure 2: Purity of the human titin fragments and binding affinities with human E3-ligases. (A) After the purification process titin fragments were controlled by 10% SDS-PAGE to detect potential degradation or contamination by other proteins. (B) Ratio of input and bound signal from the Western blot analysis for all experimental pull down preparations. Data are shown as mean ± SEM. Asterisks ($P < 0.05$ in one-way ANOVA with Dunn`s method or *t*-Test) mark statistical significance.

Supplemental figure 3

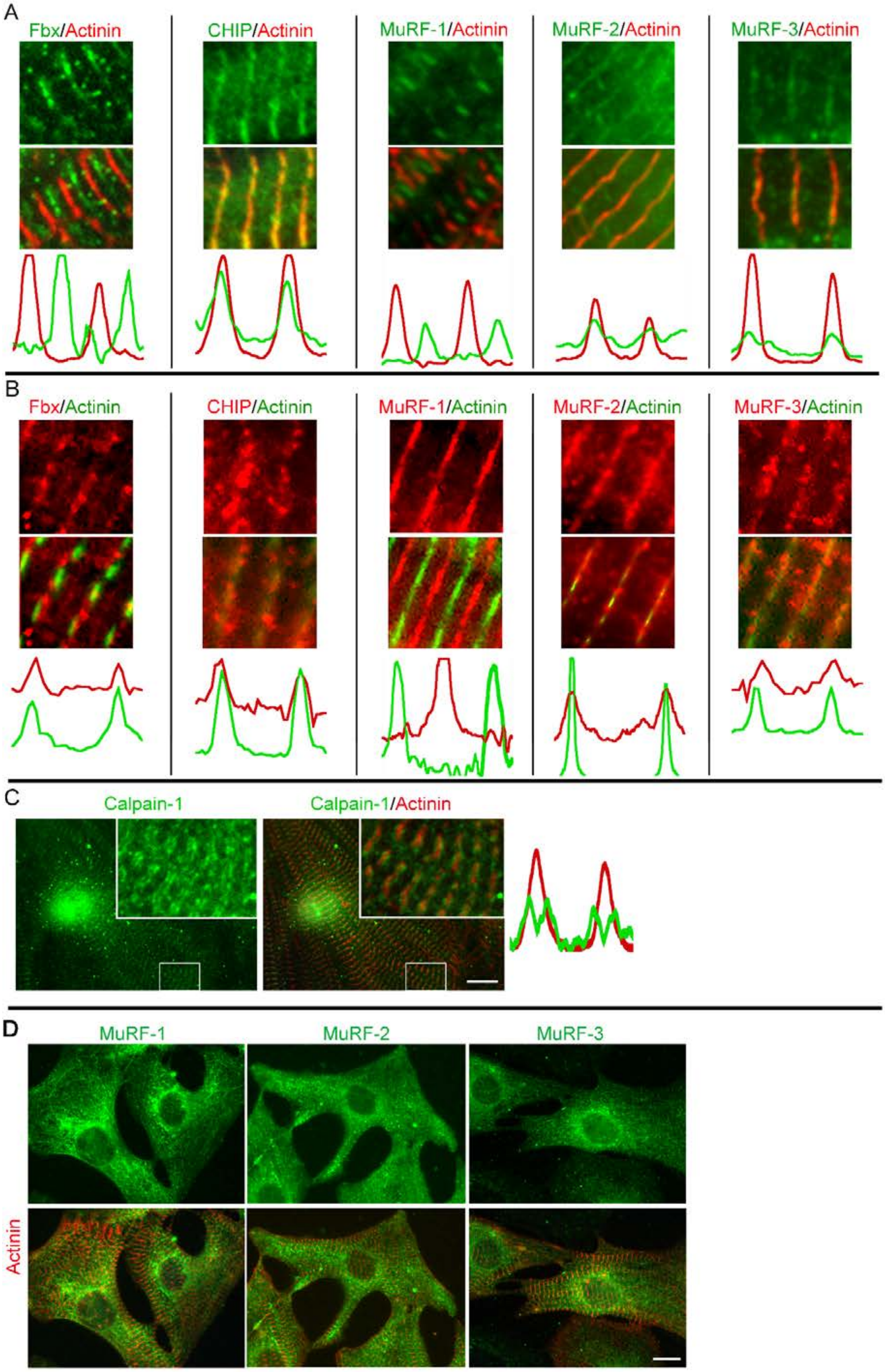

Supplemental fig. 3: Sarcomeric localization of E3-ligases and calpain in cardiomyocytes. (A) Magnifications of immunofluorescent images and antibody profiles of actinin (red) and E3-ligases (green) in ERCs. **(B)** Magnifications of immunofluorescent images and antibody profiles of actinin (green) and E3-ligases (red) in ARCs. **(C)** Immunofluorescence images and antibody profile of actinin (red) and calpain-1 (green) in ERCs. **(D)** Immunofluorescence images of MuRF-1, -2 and -3 in ERCs after 3 days in culture. Bars = 10µm.

Supplemental figure 4

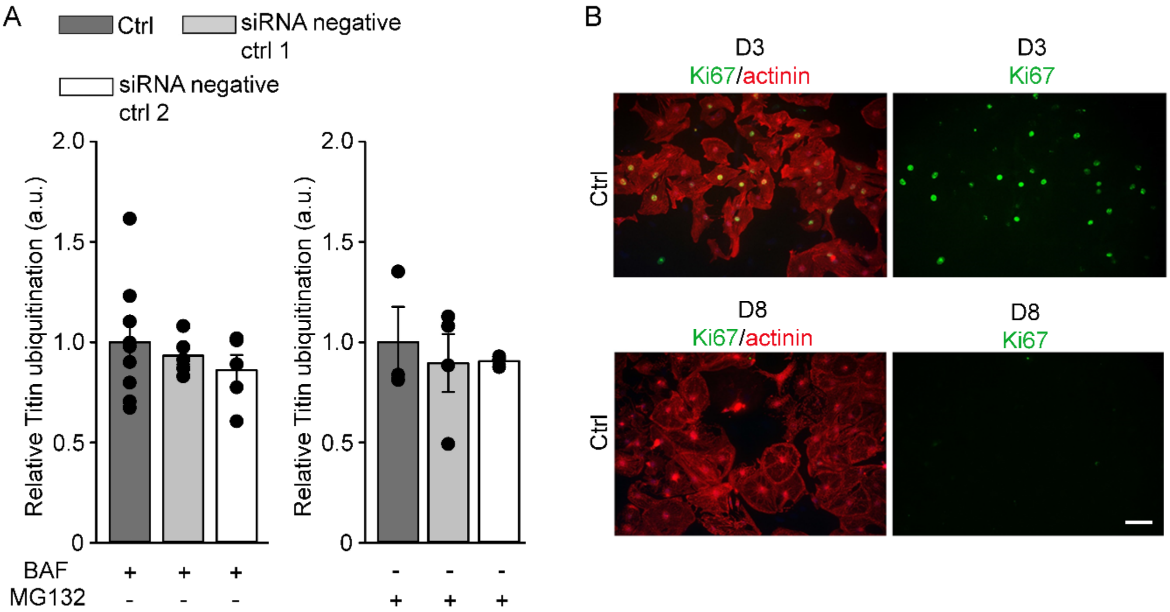

Supplemental figure 4: Relative titin ubiquitination of siRNA negative controls after inhibition of the proteasome or autophagy and proliferative capacity in ERCs. (A) siRNA negative controls do not affect titin ubiquitination after inhibition of the proteasome or autophagy. An antibody targeting titin PEVK was used in titin blot as a marker for titin loading. Data are shown as mean ± SEM. (B) Immunofluorescent stainings of Ki67 (green) and α-actinin (red) in ERC after 3 and 8 days of cultivation. BAF = bafilomycin; Ctrl = control; MG132 = proteasome inhibitor; α-Ub = anti-Ubiquitin antibody. Bar = 50 µm.

Figure 1 Autophagy and proteasomal inhibition (I)

Fig. 1B Ctrl CQ MG p62 (top), LC3 (middle), actinin (bottom)

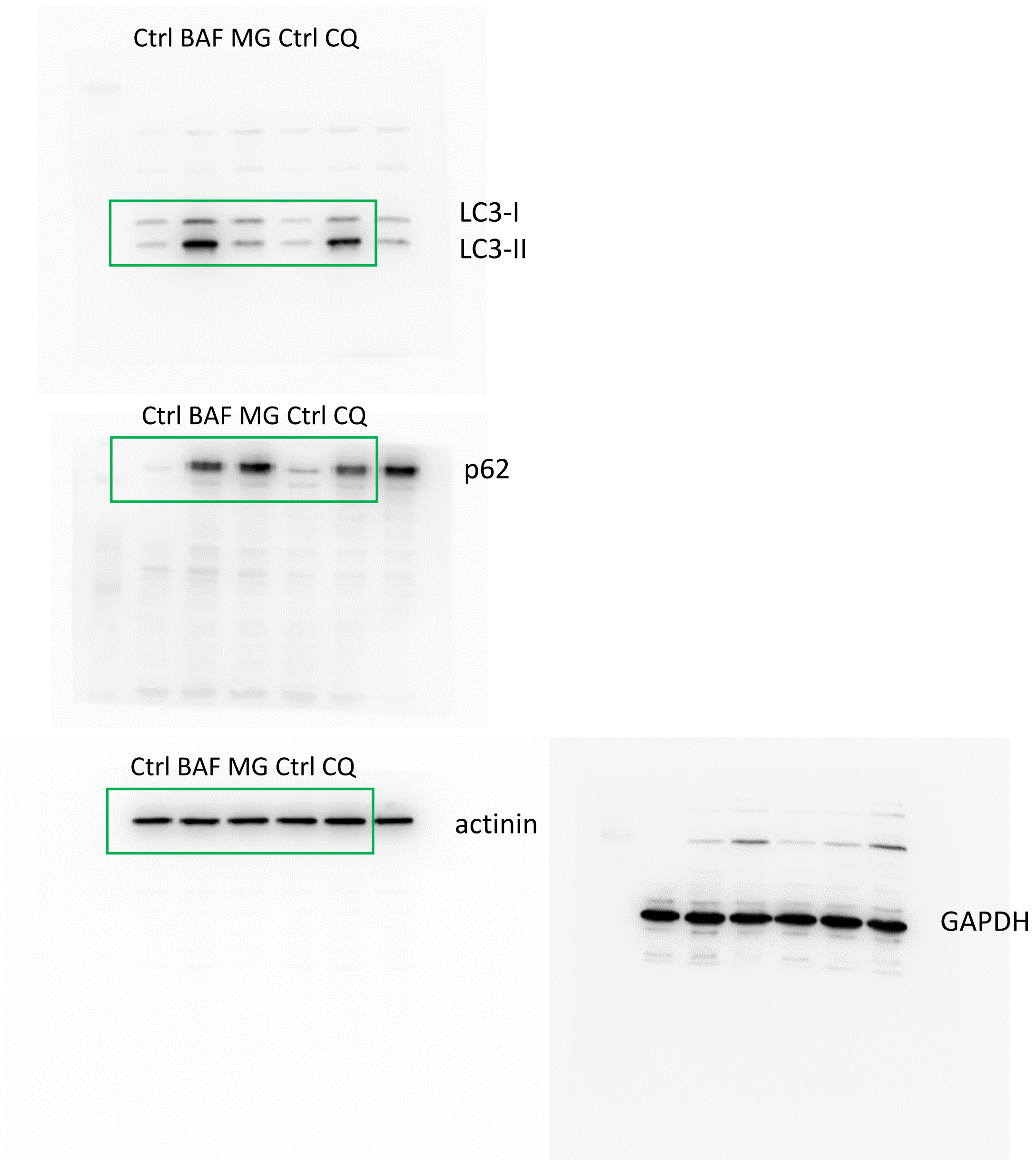

Fig. 1C Ubiquitin pan Ctrl BAF

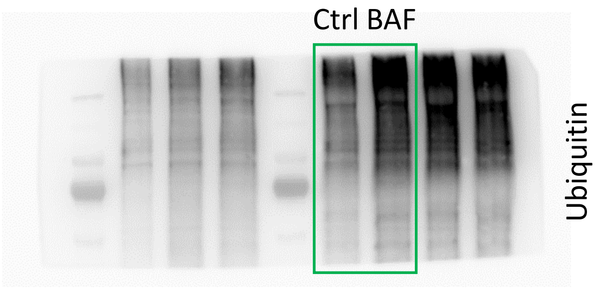

Fig. 1C Ubiquitin pan Ctrl CQ

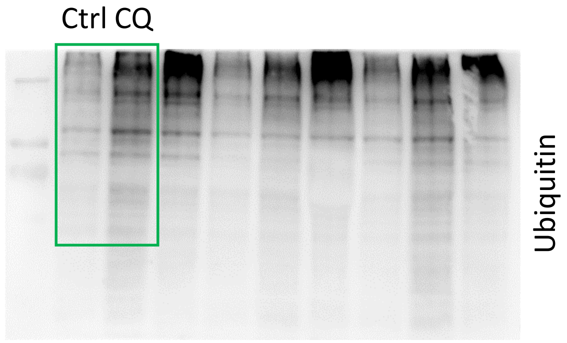

Fig. 1C Ubiquitin K63 Ctrl BAF

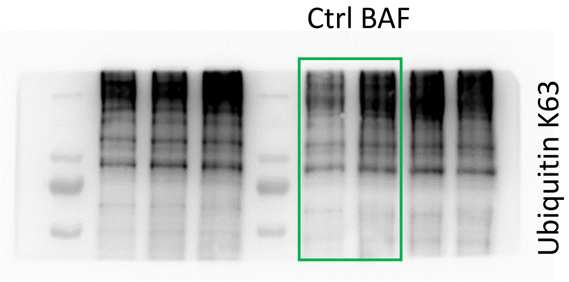

Fig. 1C Ubiquitin K63 Ctrl CQ

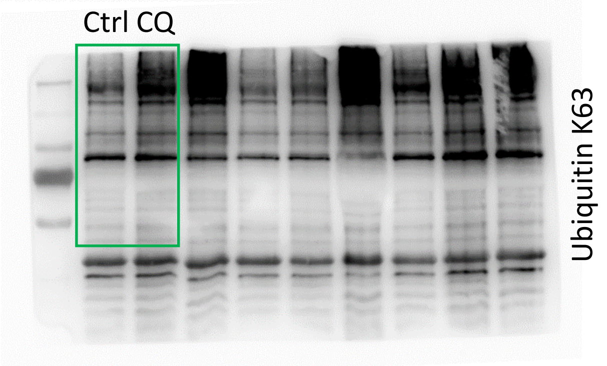

Fig. 1C GAPDH loading control

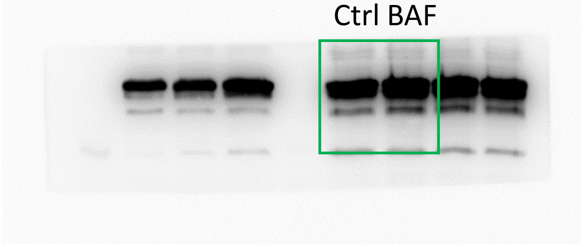

Fig. 1C GAPDH loading control

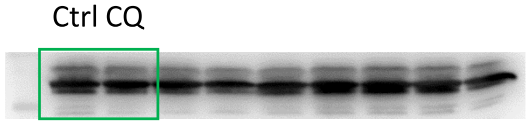

= main figure

Figure 1 Autophagy and proteasomal inhibition (II)

Fig. 1D Ubiquitin and Total Titin K63 CQ 2-24 h

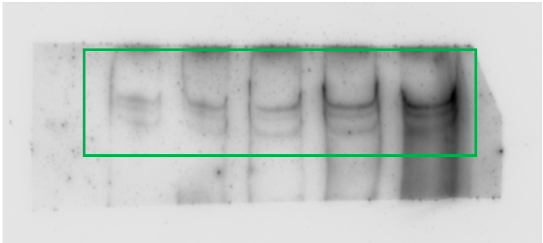

Fig. 1D Total Titin CQ 2-24 h

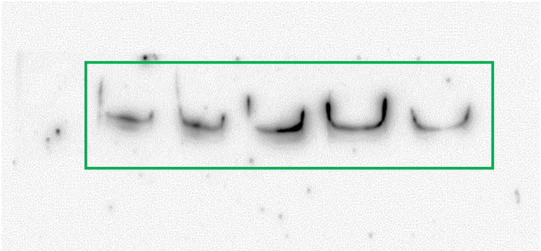

Fig. 1E Ubiquitin K63 and Total Titin BAF

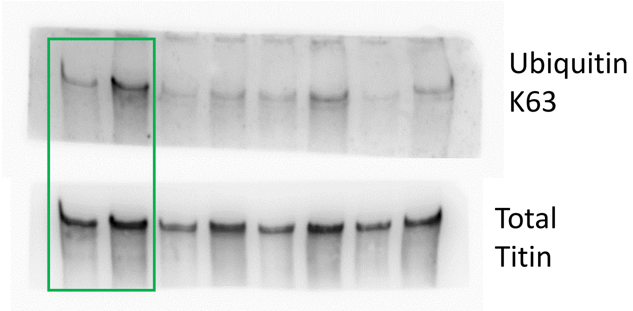

Fig. 1E Ubiquitin K63 and Total Titin CQ

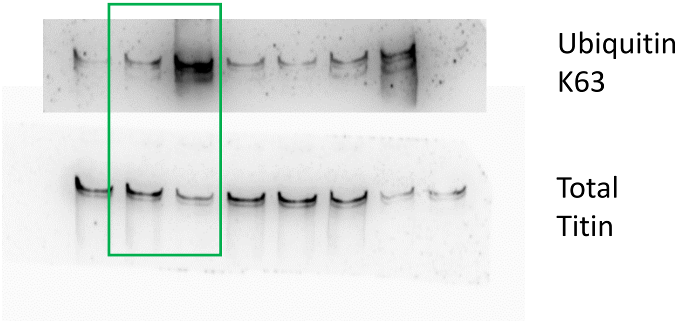

= main figure

Figure 1 Autophagy and proteasomal inhibition (III)

Fig. 1F Titin isoform and T2/T1 ratio Ctrl/CQ/BAF/MG132 2.2% SDS-PAGE Imperial protein stain

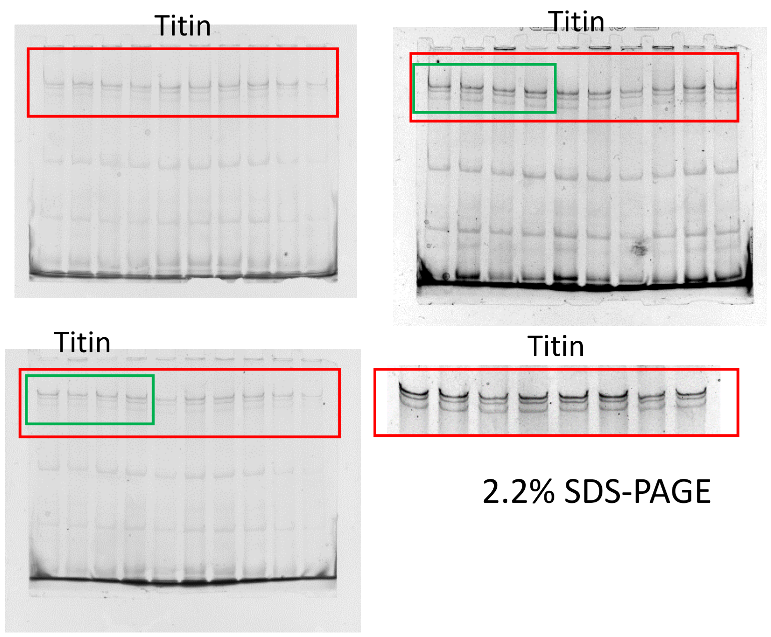

Fig. 1G titin/actinin ratio 2.2% Ctrl/BAF/MG132 SDS-PAGE Imperial protein stain

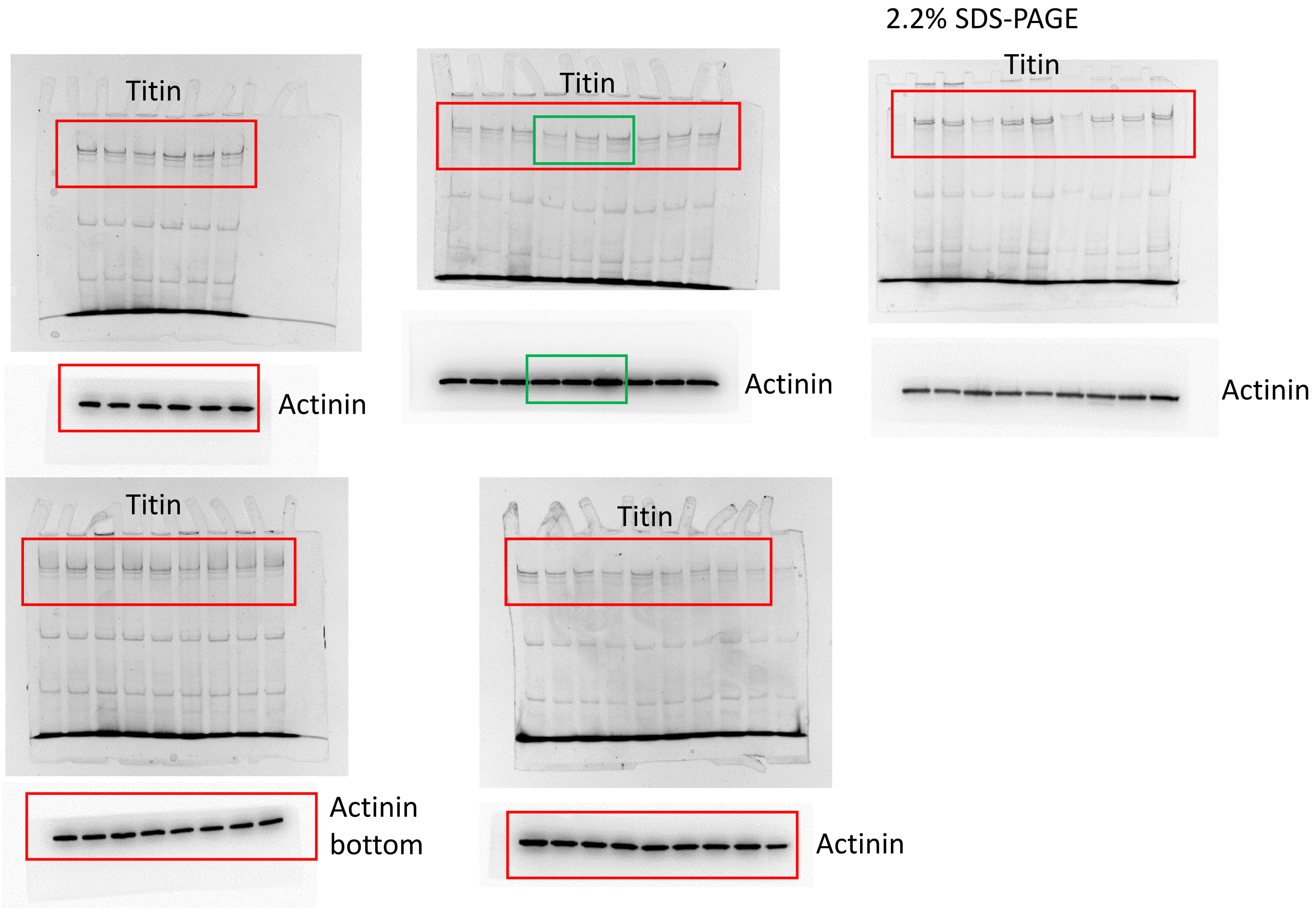

= analysis = main figure

Figure 2 pull down experiments (I)

MuRF1/MuRF-3 + N2B Input/last wash step/bound

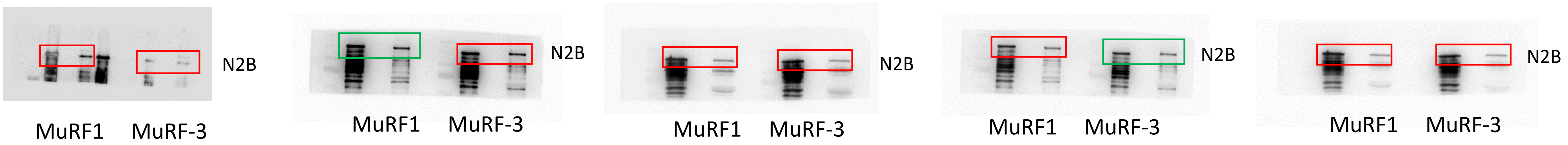

CHIP + N2B Input/last wash step/bound

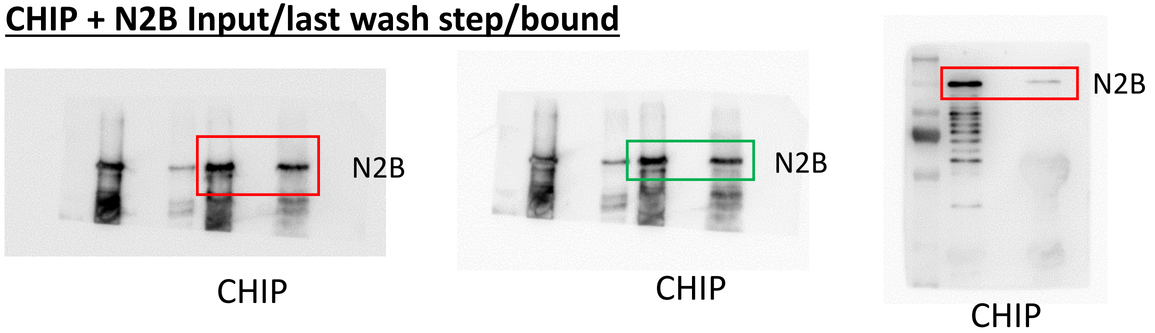

CHIP/Fbx32 + N2B Input/last wash step/bound

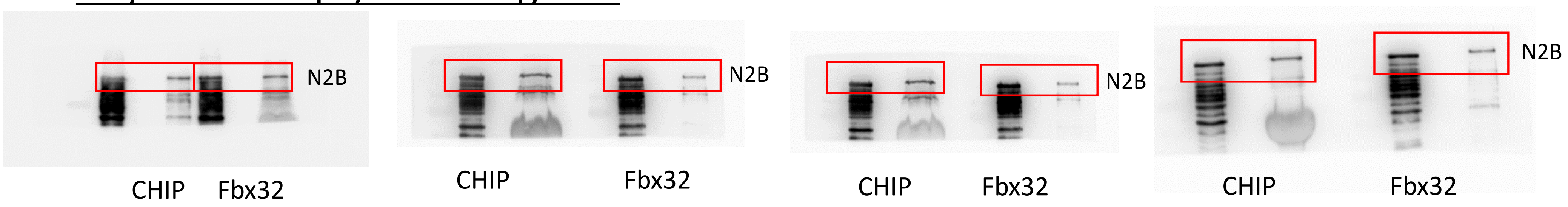

Fbx32 + N2B Input/last wash step/bound

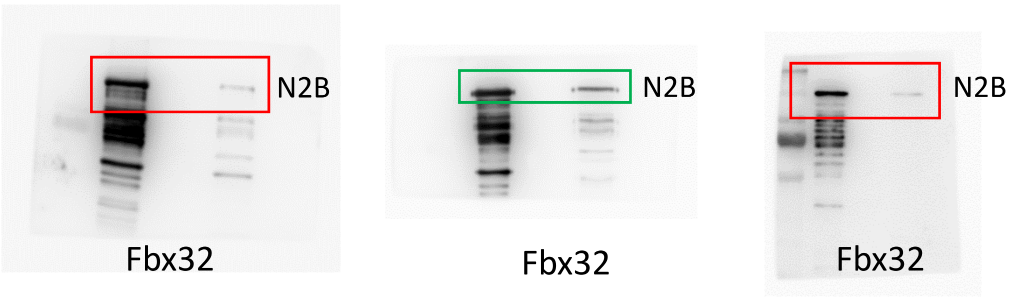

= analysis = main figure

Figure 2 pull down experiments (II)

MuRF2 + PEVK Input/last wash step/bound

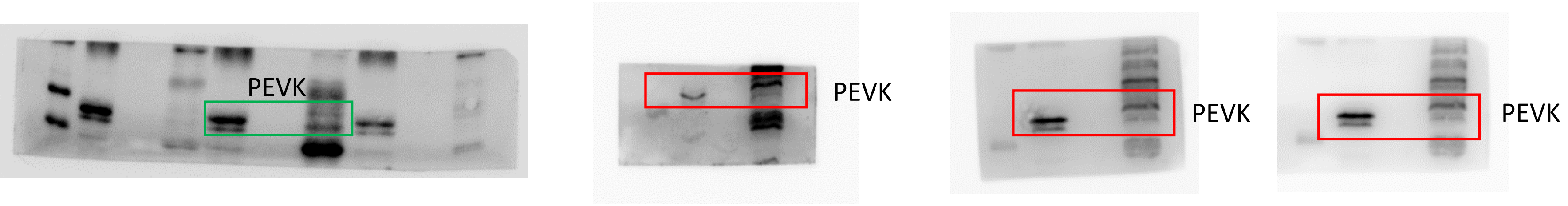

Fbx32 + N2A Input/last wash step/bound

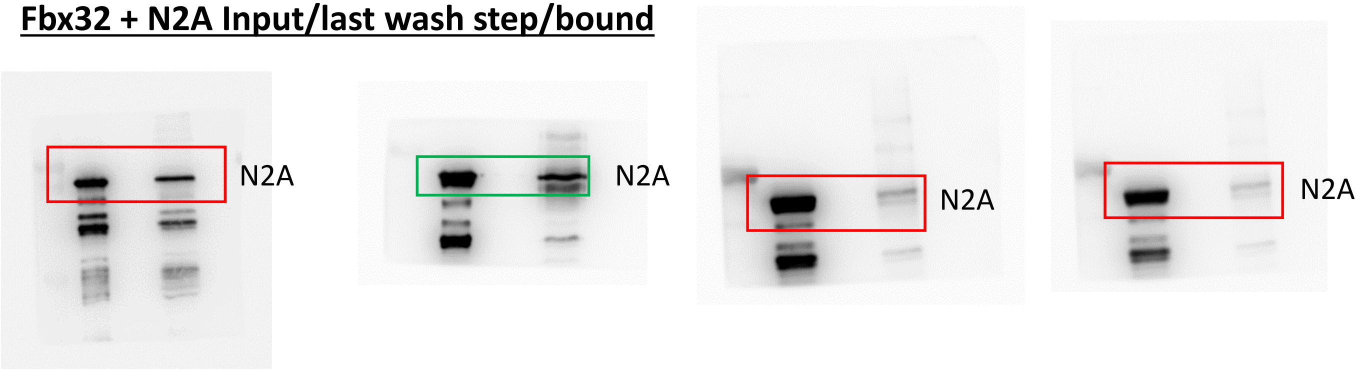

= analysis = main figure

Figure 2 pull down experiments (III)

Fbx32 + A168 Input/last wash step/bound

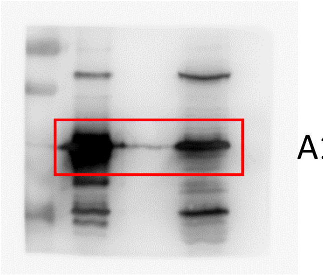

A168

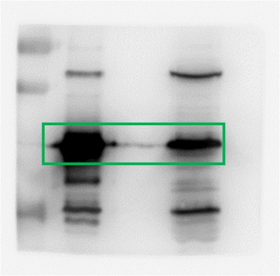

A168

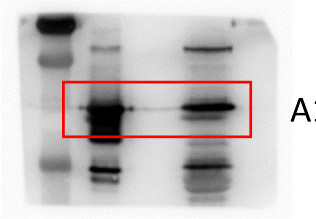

A168

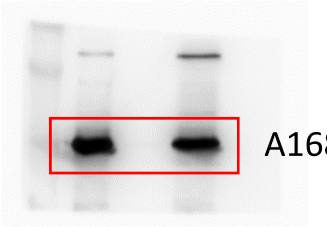

A168

CHIP + A168 Input/last wash step/bound

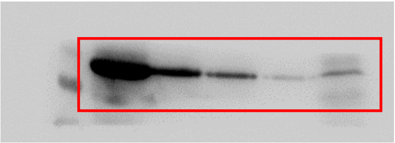

A168

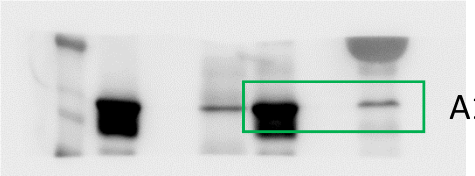

A168

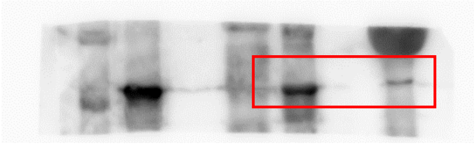

A168

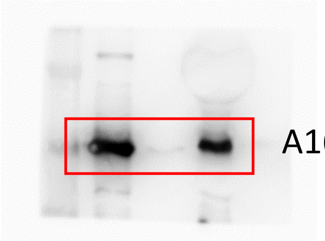

A168

= analysis = main figure

Figure 2 pull down experiments (IV)

MuRF1/MuRF-2/MuRF-3 + A168 Input/last wash step/bound

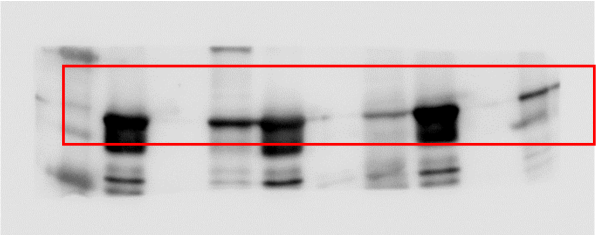

A168

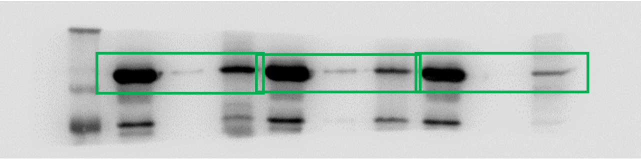

A168

MuRF1 + A168 Input/wash steps/bound

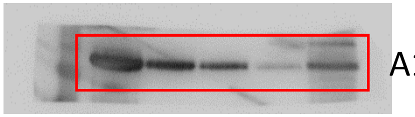

A168

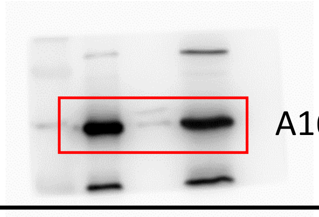

A168

MuRF2 + A168 Input/wash steps/bound

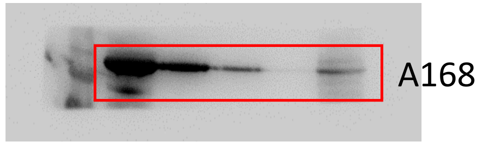

A168

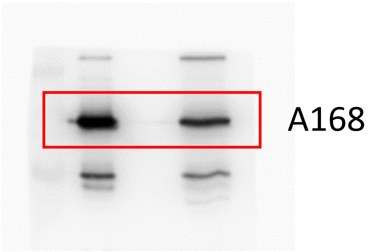

A168

MuRF3 + A168 Input/wash steps/bound

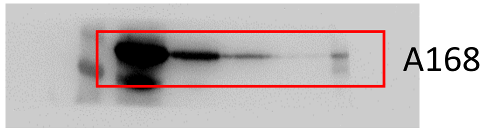

A168

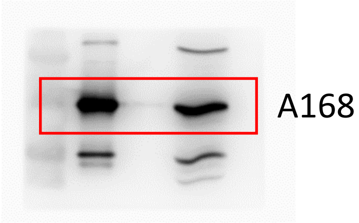

A168

= analysis = main figure

Figure 3 siRNA treatment (I)

Fig.3A E3-ligase knock down Ctrl/siRNA

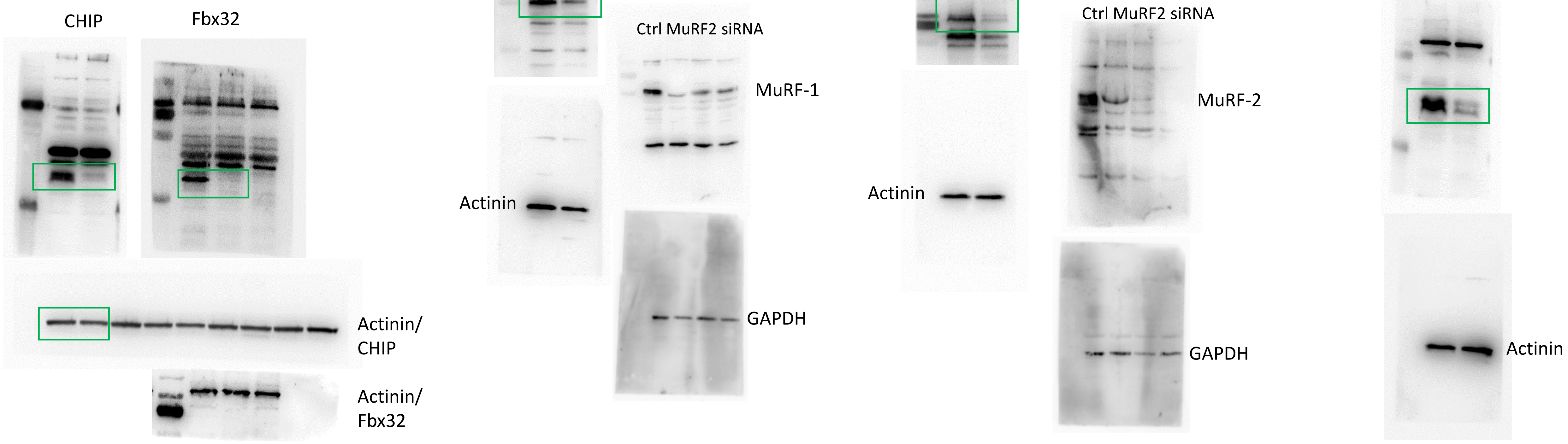

Fig.3B Titin/actinin ratio analysis

2.2% SDS-PAGE, Imperial protein stain

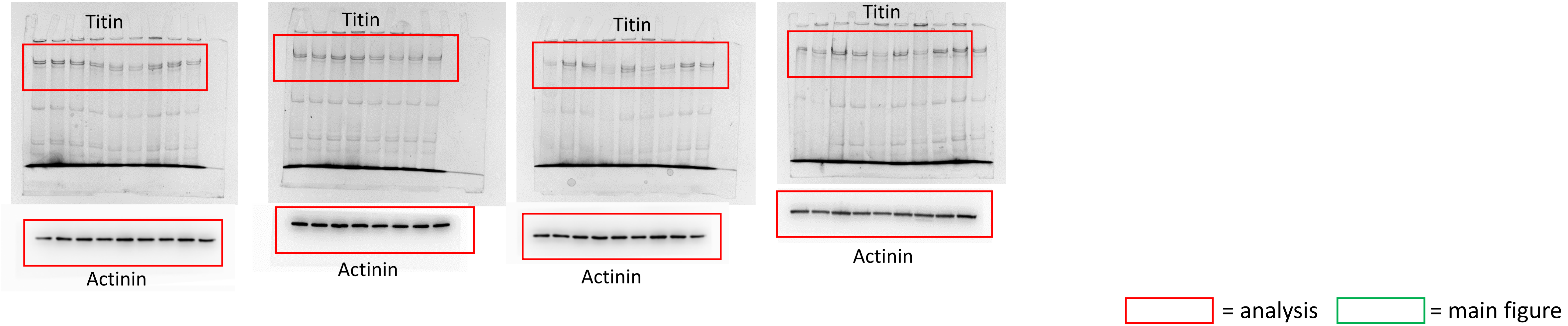

Figure 3 siRNA treatment (II)

Fig.3C MG Ubiquitin pan (main figure bottom)

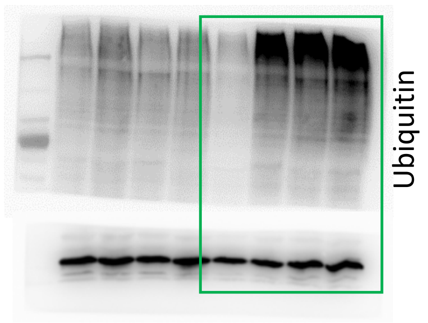

Fig.3C MG132 Ubiquitin (analysis)

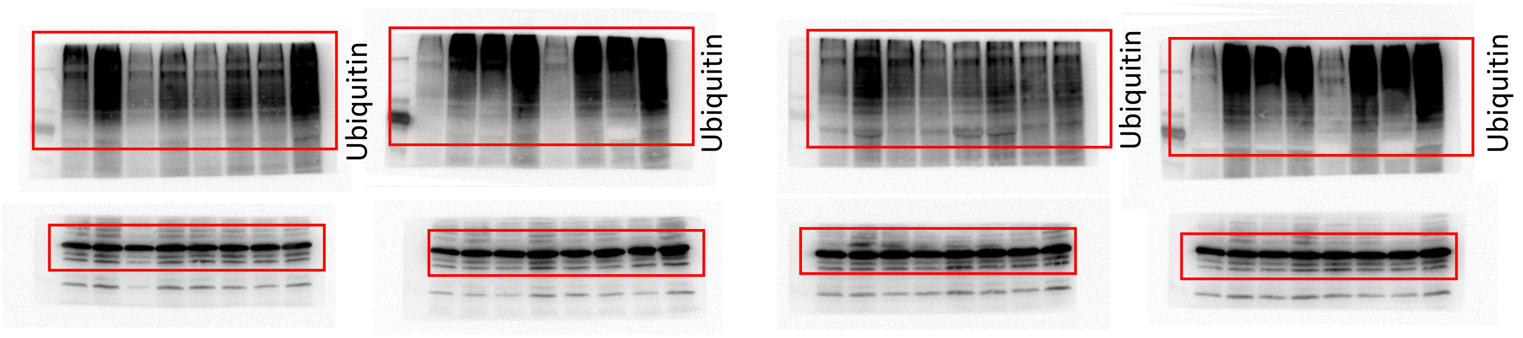

Fig. 3C BAF Ubiquitin pan (main figure top)

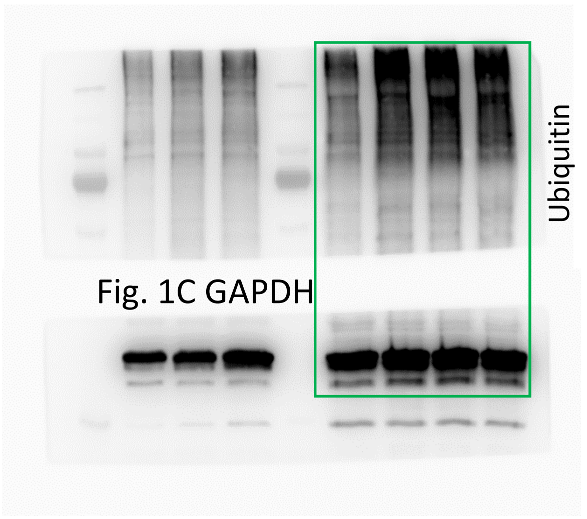

Fig.3C BAF Ubiquitin (analysis)

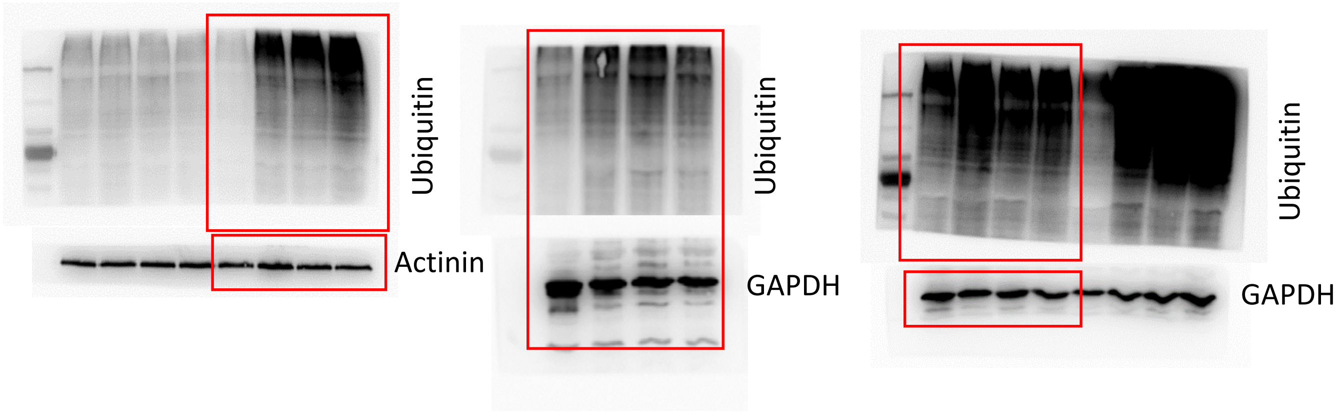

= main figure = analysis

Figure 3 siRNA treatment (III)

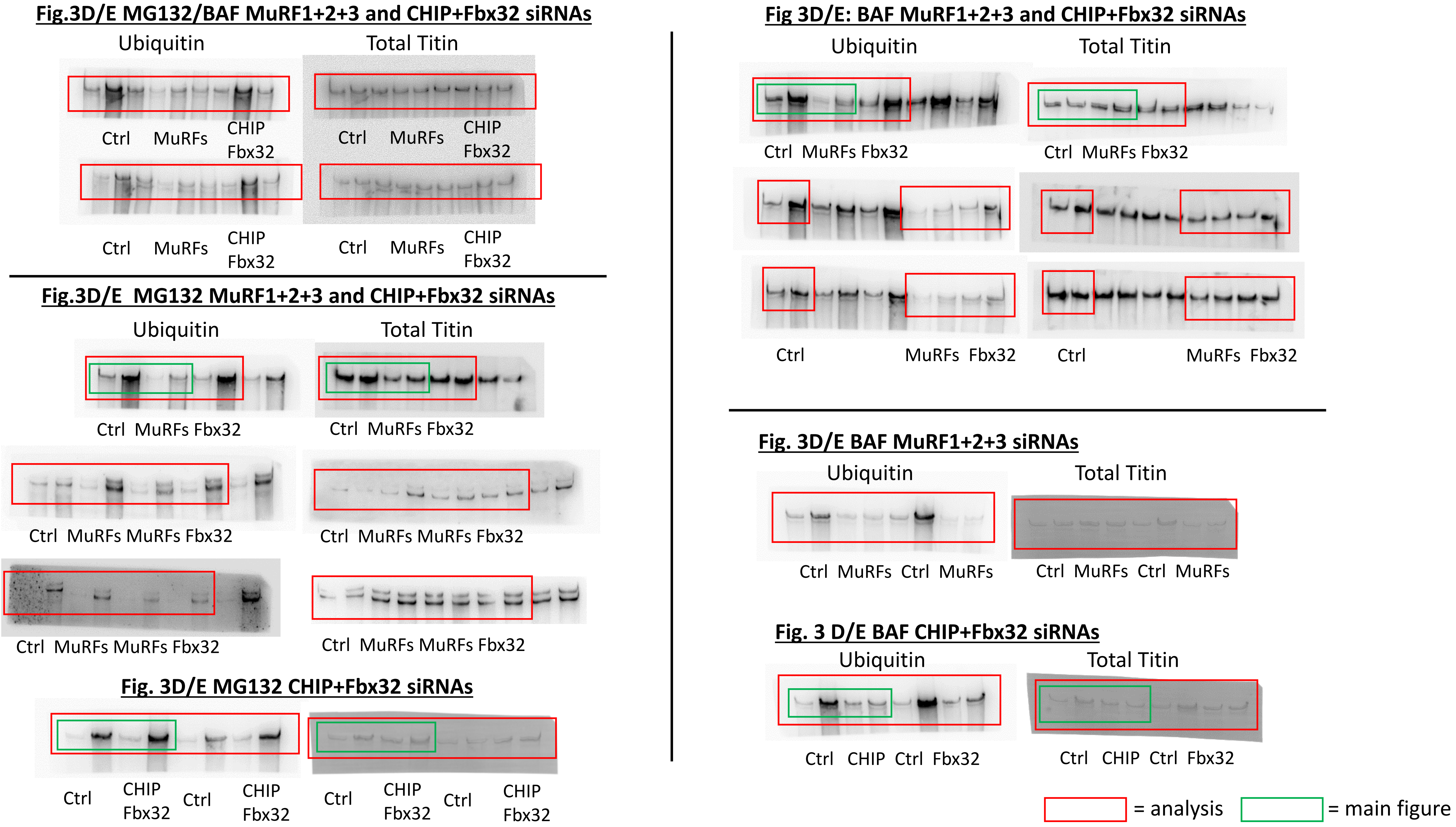

Figure 4: siRNA treatment (I)

Fig. 4A Ctrl/MuRF 1/2/3 single siRNAs Ctrl/BAF

Ubiquitin Total Titin

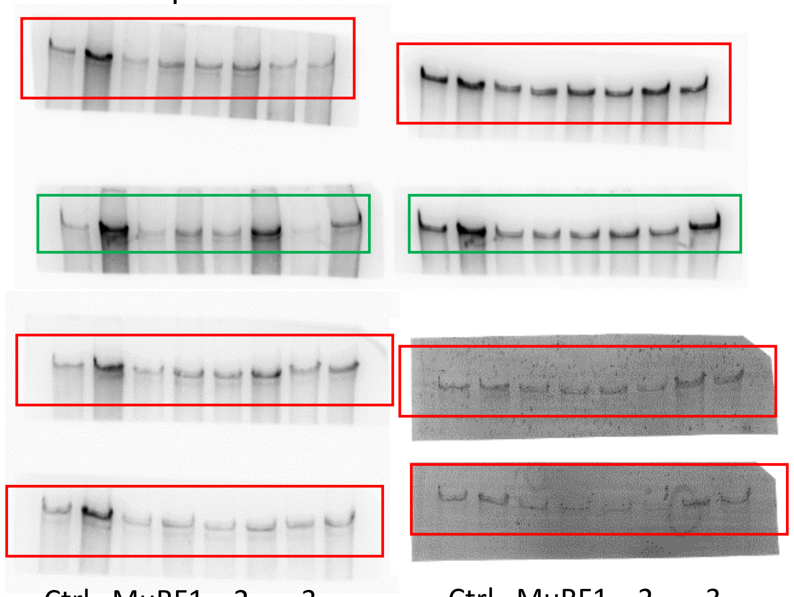

Ctrl MuRF1 2 3 Ctrl MuRF1 2 3

Fig. 4A Ctrl/MuRF 1/2/3 single siRNAs Ctrl/MG132

Ubiquitin Total Titin

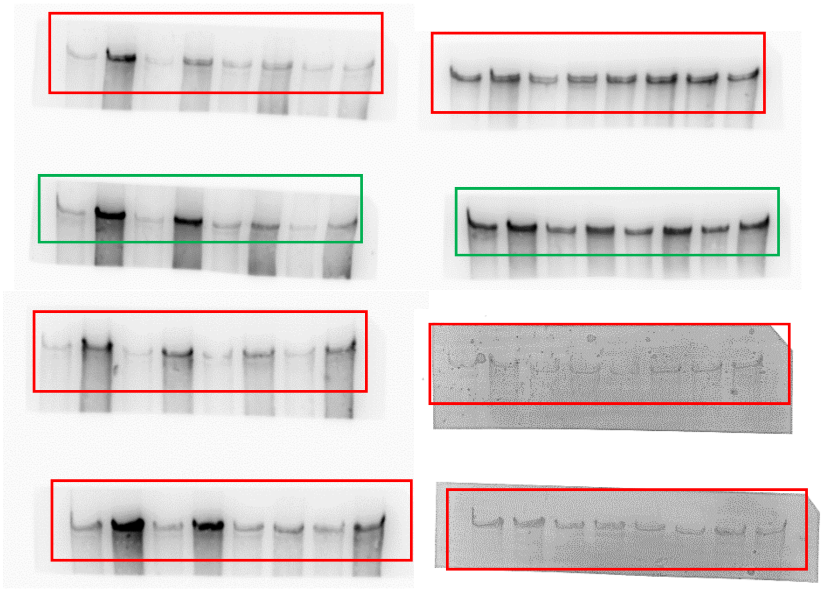

Ctrl MuRF1 2 3 Ctrl MuRF1 2 3

= analysis = main figure

Figure 4: siRNA treatment (II)

Fig. 4B Ctrl vs CHIP siRNA Ctrl/BAF/MG132

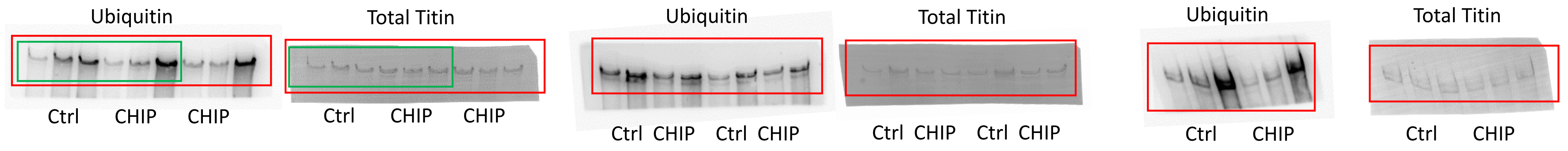

Fig. 4B Ctrl vs CHIP or Fbx32 siRNA Ctrl/BAF/MG132

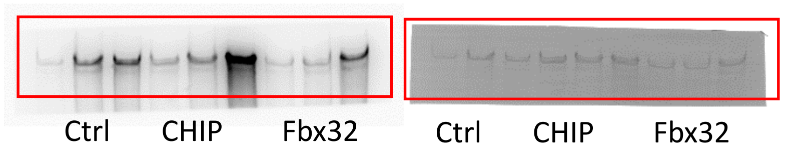

Fig. 4B Ctrl vs Fbx32 siRNA Ctrl/BAF/MG132

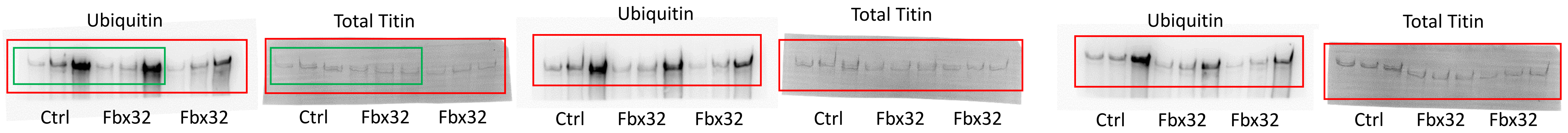

= main figure = analysis
